# Cu^2+^ and Zn^2+^ Ions Affecting Biochemical Paths and DNA Methylation of Rye (*Secale cereale* L.) Anther Culture Influencing Plant Regeneration Efficiency

**DOI:** 10.3390/cells14151167

**Published:** 2025-07-29

**Authors:** Wioletta Monika Dynkowska, Renata Orłowska, Piotr Waligórski, Piotr Tomasz Bednarek

**Affiliations:** 1 Plant Breeding and Acclimatization Institute-National Research Institute, Radzików, 05-870 Błonie, Poland; w.dynkowska@ihar.edu.pl; 2 The Franciszek Górski Institute of Plant Physiology, Polish Academy of Sciences, Niezapominajek 21, 30-239 Kraków, Poland; p.waligorski@ifr-pan.edu.pl

**Keywords:** rye anther culture, DNA sequence context, albino plants, GPRE, copper, zinc, polyamines, phytohormones, SAM, GSH

## Abstract

Rye regeneration in anther cultures is problematic and affected by albino plants. DNA methylation changes linked to Cu^2+^ ions in the induction medium affect reprogramming microspores from gametophytic to sporophytic path. Alternations in S-adenosyl-L-methionine (SAM), glutathione (GSH), or β-glucans and changes in DNA methylation in regenerants obtained under different in vitro culture conditions suggest a crucial role of biochemical pathways. Thus, understanding epigenetic and biochemical changes arising from the action of Cu^2+^ and Zn^2+^ that participate in enzymatic complexes may stimulate progress in rye doubled haploid plant regeneration. The Methylation-Sensitive Amplified Fragment Length Polymorphism approach was implemented to identify markers related to DNA methylation and sequence changes following the quantification of variation types, including symmetric and asymmetric sequence contexts. Reverse-Phase High-Pressure Liquid Chromatography (RP-HPLC) connected with mass spectrometry was utilized to determine SAM, GSH, and glutathione disulfide, as well as phytohormones, and RP-HPLC with a fluorescence detector to study polyamines changes originating in rye regenerants due to Cu^2+^ or Zn^2+^ presence in the induction medium. Multivariate and regression analysis revealed that regenerants derived from two lines treated with Cu^2+^ and those treated with Zn^2+^ formed distinct groups based on DNA sequence and methylation markers. Zn^2+^ treated and control samples formed separate groups. Also, Cu^2+^ discriminated between controls and treated samples, but the separation was less apparent. Principal coordinate analysis explained 85% of the total variance based on sequence variation and 69% of the variance based on DNA methylation changes. Significant differences in DNA methylation characteristics were confirmed, with demethylation in the CG context explaining up to 89% of the variance across genotypes. Biochemical profiles also demonstrated differences between controls and treated samples. The changes had different effects on green and albino plant regeneration efficiency, with cadaverine (Cad) and SAM affecting regeneration parameters the most. Analyses of the enzymes depend on the Cu^2+^ or Zn^2+^ ions and are implemented in the synthesis of Cad, or SAM, which showed that some of them could be candidates for genome editing. Alternatively, manipulating SAM, GSH, and Cad may improve green plant regeneration efficiency in rye.

## 1. Introduction

The rye’s low androgenic response and regeneration efficiency [1,2] are closely linked to the genotype, exhibiting a significant variation between each other, and even a combination of optimally performing conditions for one genotype can remain without a positive effect in recalcitrant genotypes [3]. The androgenic response and plant regeneration start with reprogramming microspores from gametophytic to sporophytic fate, involving biochemical changes, e.g., activation of lignin biosynthesis during the early stages of microspore embryogenesis [4], regulated by metal ions acting as cofactors in metalloenzymes (copper ions in laccases) [5]. Changes in biochemical paths may result in modification of epigenetic factors such as DNA methylation [6] or alterations in histone modifications, resulting in transcription pattern variation that assists cell reprogramming and plant regeneration [7,8]. The process proceeds through the formation of androgenic embryos and ends with the regeneration of haploid plants. Green plant regeneration may force researchers to develop protocols for a given genotype to maximize regeneration potential for a given line [9]. Moreover, androgenic response and regeneration efficiency depend, among others, on well-tuned tissue culture methods.

In rye, obtaining haploid plants or doubled haploids is usually achieved by anther culture, where anthers at the appropriate developmental stage of microspores are plated onto solid media [10], or in isolated microspore cultures, where microspores are released from the anthers by mechanical crushing and then suspended in liquid media [11]. Supplementation of in vitro culture media with metal ions is often recommended [12,13,14,15]. Among many metal ions used as ingredients of in vitro media, copper and zinc are widely explored as they are cofactors of biochemical pathways. Copper ions are cofactors of complex IV, called cytochrome *c* oxidase, in the electron transport chain (ETC) [16]. This complex is involved in the transport of electrons to complex V, where adenosine triphosphate (ATP), the most prominent intracellular energy carrier, is formed [17]. Copper deficiencies can disrupt electron flow function and, at the same time, interfere with ATP production, thereby affecting cell energetics and function. ATP deficiency can affect the Yang cycle, which produces S-adenosyl-L-methionine (SAM), the primary donor of methyl groups [18], impairing the process of DNA methylation [19] and thereby affecting the reprogramming of microspores from a gametophytic to sporophytic fate [20] through methylation-dependent regulation of gene expression. The disruption in SAM production caused by the lack of ATP may also affect glutathione (GSH) formation because SAM is involved in the conversion of homocysteine to cysteine (transsulfuration pathway), which is the precursor of GSH [21].

In previous studies, the positive effect of copper added to the induction medium in isolated microspore culture or in vitro anther culture has been reported in tetraploid wheat [22,23], millet [24], barley [15,25], sorghum [26], and rice [27]. The presence of copper in the induction medium is also one of the determinants of the androgenic response in rye [28,29]. Furthermore, studies on obtaining barley and triticale regenerants have shown an essential role of copper in the regeneration of green plants in anther culture [30]. Copper ion was a key factor linking the Krebs cycle via ETC to the Yang cycle and SAM production to DNA methylation (epigenetic processes) and to GSH biosynthesis [30,31].

Furthermore, Cu^2+^ may act as a cofactor in copper-containing amine oxidase (CuAOx), participating in the oxidative deamination of polyamines (PA), mainly putrescine (Put), and cadaverine (Cad) and less efficiently spermidine (Spd) and spermine (Spm) at the primary amino groups with producing ammonia, hydrogen peroxide (H_2_O_2_) and an amino aldehyde as a byproduct in the polyamine catabolism [32,33,34]. Generated H_2_O_2_ may act as a signaling molecule, influencing cellular reprogramming and embryogenesis [35]. CuAOx also impacts cell wall remodelling by producing H_2_O_2_, which participates in forming lignin and cross-linking cell wall constituents [36]. The reprogramming of microspore development from gametophytic to sporophytic, in turn, depends on cell wall remodelling [37]. Therefore, CuAOx might indirectly help microspore reprogramming succeed. The significance of spermidine and putrescine was described for carrot anther cultures [38]. The two polyamines increased embryo formation, and when added to the regeneration medium, an increase in plant regeneration from androgenic embryos was documented. Furthermore, spermidine stimulated gametic embryogenesis in the *Citrus clementina*, cv Nules, whereas putrescine failed to affect embryo production [39]. The significance of other polyamines on green plant regeneration efficiency (GPRE) is not apparent; however, their biosynthesis is linked to the Yang cycle [40], which depends on Cu^2+^, and, assuming the importance of other polyamines in plant regeneration, their role should be investigated.

Zinc is similarly essential for plant cellular processes [41,42]. Zinc deficiency has been linked to chlorophyll loss in wheat regenerants [43], and the element is crucial for many transcription factors, especially those with zinc-finger domains [44,45,46], such as *OsLSD1*, which regulates callus differentiation in rice [47]. Zinc addition has improved embryogenesis and regeneration in barley [13] and rice [48], though results may vary by species, as seen in oats [12]. Zinc supports tryptophan synthesis [49] via tryptophan synthase, which contributes to indole-3-acetic acid (IAA) production [50], a key hormone in cell division and differentiation [51]. Zinc may affect IAA both through the biosynthesis of tryptophan [52] and indirectly by modulating ROS scavenging systems that prevent IAA degradation [53]. However, no confirmed link between Zn^2+^ and the biosynthesis of other phytohormones used in tissue culture has been found.

Metal ions are vital cofactors for enzymatic reactions, forming complexes with proteins such as Cu-Zn superoxide dismutase (Cu/ZnSOD) [54] and cytochrome *c* oxidase (COX) [55]. Deficiencies, excesses, or absences of these ions disrupt cellular homeostasis [56,57] and impair biochemical pathways [58,59], obstructing the crucial reprogramming from gametophytic to sporophytic development [60]. This disruption directly undermines the regeneration efficiency of green plants [43,61].

Based on available data, it is being postulated that Cu^2+^ and Zn^2+^ ions, as ingredients added to in vitro regeneration medium, may affect biochemical pathways such as the Krebs and Yang cycles, influencing SAM and possibly polyamines (Cad, Put, Spd, Spm) and phytohormones (IAA, isopentenyl adenine: IPA; abscisic acid: ABA; salicylic acid: SA; jasmonic acid: JA; *trans*-zeatin: tZ; *cis*-zeatin: cZ; *trans*-zeatin riboside: tZR; *cis*-zeatin riboside: cZR) syntheses, which impact plant regeneration. Furthermore, microspore reprogramming may induce changes in DNA methylation patterns that could be investigated via DNA marker systems. Varying metal ion ingredients can change plant regeneration conditions towards increased/decreased regeneration of green plants via anther cultures in rye. Changes in the levels of metabolites such as SAM, GSH, glutathione disulfide (GSSG), and phytohormones can be studied by Reverse-Phase High-Pressure Liquid Chromatography with mass spectrometry (RP-HPLC-MS). In contrast, changes in polyamines can be studied by RP-HPLC with a fluorescence detector (RP-HPLC-FLD).

The study aims to verify whether Cu^2+^ and Zn^2+^ impact biochemical paths and DNA methylation patterns of regenerants derived via anther culture, affecting GPRE in rye, and which of the factors, biochemical or (epi)genetic, is the most influential.

## 2. Materials and Methods

### 2.1. Donor Plants’ Growing Conditions and Plant Regeneration

**Figure 1:**
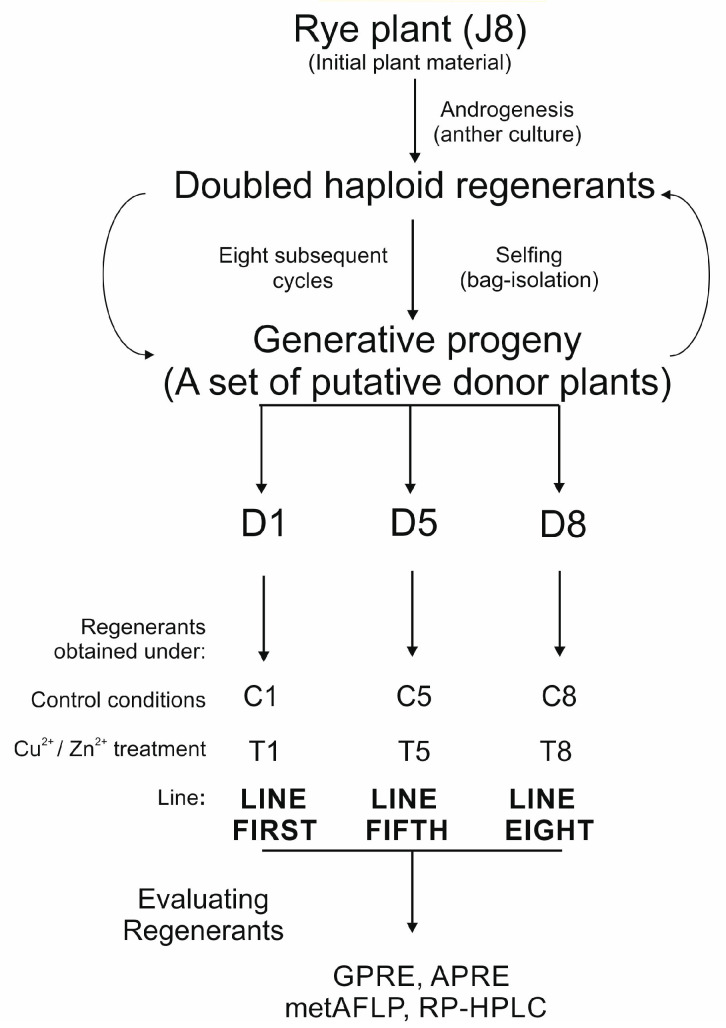
Plant material evaluation and analysis. Randomly chosen plants representing cultivar J8 were subjected to in vitro anther culture. Regenerants were selfed, and randomly chosen progeny were employed for the next round of in vitro anther cultures, following regenerant selfing and derivation of progeny. Eight such cycles were conducted, and three plants representing three homozygous lines (first, fifth, and eighth) with stable androgenic efficiency were chosen for further analysis. The generative progeny of selfed doubled haploids’ rye regenerant obtained in anther cultures from microspores constituted three donor plants: D1, D5, and D8. These donor plants were used as a source of anthers to obtain regenerants under the varying concentrations of Cu^2+^ and Zn^2+^ present in the induction medium; C reflected control conditions and T tested approach. The regenerants for each trial were counted, and green plant regeneration efficiency (GPRE) and albino plant regeneration efficiency (APRE) were evaluated. MetAFLP and RP-HPLC analyses were performed for each regenerant, and the evaluated data were implemented for statistical analysis.

The details of plant material evaluation and subsequent analyses are illustrated in Figure 1.

A 1.5% aqueous sodium hypochlorite solution was used to surface-sterilize rye plants’ mature dry seeds for 10 min. The seeds were then rinsed three times with distilled water, put on wet filter paper in Petri dishes, and left for 24 h at 4 °C and then for another 24 h at room temperature. Sprouted seeds were placed in seedling trays filled with a 3:1 mixture of soil and sand and subsequently were cultivated in cylindrical pots with a diameter of 26 cm, a height of 23 cm, and an estimated volume of approximately 23 L. The donor plants (D1, D5, D8) were cultivated in a growth chamber under carefully monitored conditions at 14 °C/10 °C (16 h/8 h; light/dark; light intensity 190 µE m^−2^ s^−1^ maintained with high-pressure sodium lamps) until the fourth leaf was fully developed. The seedlings were then vernalized for eight weeks at 4 °C with a short photoperiod (8 h/16 h; light/dark) and 20 µE m^−2^ s^−1^ of light intensity. After vernalization, plants were potted into pots filled with a soil–sand combination and grown in a growth chamber at 16 °C/12 °C (16 h/8 h; light/dark). Tillers of donor rye plants were ready to harvest when the majority of the microspores were in the mid- to the uninucleate stage. The developmental stage of microspores was estimated by acetic carmine staining. Collected tillers were wrapped in plastic bags and stored for 14 days at 4 °C in the dark in jars with water. Anthers were collected from cut spikes that had been sterilized by soaking in 70% ethanol for 1 min, followed by soaking in 1.5% sodium hypochlorite for 20 min, and then washed four times with sterile deionized water. After sterilizing the spikes, the rye anthers were removed, placed on 0.4 M mannitol solution containing cefotaxime (0.1 g L^−1^), and left at 6 °C for 7 days (one spike per plate). Then, under sterile conditions, the anthers were placed on a semi-solid induction medium (IM) 190-2 [62] with 90 g L^−1^ maltose and 438 mg L^−1^ glutamine supplemented with 2 mg L^−1^ 2,4-dichlorophenoxyacetic acid (2,4-D) and 0.5 mg L^−1^ kinetin (control condition). The tested in vitro conditions included supplementation of the 190-2 with copper (II) ions (T1, T5; [Cu^2+^] = 5 µM for anthers from D1 and D5 donor plants) and zinc (II) ions (T8; [Zn^2+^] = 150 µM for anthers from D8 plant) (Figure 1). Half of the anthers from one spike were plated on the control induction medium (C: C1, C5, C8) and half on the tested induction medium (T: T1, T5, T8). Anthers were incubated at 23 °C in the dark on all induction media. The embryos, calli, and embryo-like structures were moved onto a regeneration medium 190-2 [62] after approximately 35 days. The regeneration medium was supplemented with 1.5 mg L^−1^ kinetin and 0.5 mg L^−1^ naphthalene acetic acid (NAA). The incubation was carried out at 26 °C with a 16 h/8 h light/dark photoperiod and a light intensity of 50 µE m^−2^ s^−1^ on regeneration medium. Glass flasks filled with N6I rooting medium [63] and 2 mg L^−1^ IAA were used to hold the green plants. The developed plantlets were vernalized at 4 °C for eight weeks after being placed in containers filled with a soil–sand mixture. Plants were grown in a greenhouse following vernalization. Three sets of regenerants were finally obtained: line first-regenerants derived from the D1-donor plant, line fifth-regenerants derived from the D5-donor plant, and line eighth-regenerants derived from the D8-donor plant (Figure 1). In mature rye regenerants, spontaneous chromosome doubling was determined by assessing the fertility of regenerated plants, specifically their seed-setting ability. In addition, plant morphology was assessed by considering plant growth, leaf shape, colour, and width, as well as tillering mode and spike number, in comparison to the donor plants. In each trial, the number of green regenerants and albino regenerants obtained per 100 plated anthers was estimated and referred to as green plant regeneration efficiency (GPRE) or albino plant regeneration efficiency (APRE). GPRE and APRE provide a measure of the efficiency of regenerant production of rye in vitro cultures.

### 2.2. DNA Extraction and Methylation-Sensitive Amplified Fragment Length Polymorphism (metAFLP) Procedure

Tissue from young rye leaves of donor and regenerant plants was crushed in liquid nitrogen. The Plant DNeasy MiniPrep Kit (Qiagen, Hilding, Germany) was used to extract the genomic DNA. A spectrophotometer operating at λ_1_ = 260 nm and λ_2_ = 280 nm wavelengths was used to determine the concentration and purity of the DNA. DNA samples were examined for integrity using ethidium bromide staining in 1.2% agarose gel electrophoresis. Ultimately, 500 ng of DNA was prepared for each restriction reaction.

The metAFLP method’s subsequent steps were carried out by earlier publications [64], although the process was modified somewhat [65,66]. In brief, the methylation variant of the AFLP technique, which is based on cutting DNA fragments with restriction enzymes and amplifying the resulting fragments, utilises two pairs of enzymes, Acc65I, MseI and KpnI, MseI [64].

The sequence-based markers were generated using the metAFLP approach with KpnI/MseI digests. KpnI is insensitive to DNA methylation at its recognition site and in adjacent regions.

In contrast, the Acc65I/MseI platform uses Acc65I, an isoschizomer that recognizes the same restriction site as KpnI but is sensitive to methylation at and near this site. This makes the Acc65I/MseI platform suitable for detecting both sequence variation and DNA methylation events.

To extract information specifically related to DNA methylation, virtual methylation markers were derived by comparing the Acc65I/MseI and KpnI/MseI datasets. Markers that were either present or absent in both datasets were considered sequence-related and coded as ‘0’, while markers that appeared in only one dataset were attributed to methylation changes and coded as ‘1’. Based on this logic, and assuming that KpnI/MseI and Acc65I/MseI markers were juxtaposed, a methylation matrix could be effectively constructed.

Two DNA samples containing 500 ng of genomic DNA were prepared for each plant under test. Acc65I/MseI and KpnI/MseI, two pairs of restriction enzymes, were used to digest these materials concurrently. Following their ligation, synthesized oligonucleotides (adapters) were affixed to the DNA fragments.

For the preselective PCR, 2.5 µL of the ligation product was sampled after diluting it with water (1:3). DNA fragments were amplified while preselective primers were present. The preselective amplification product was diluted with water (1:20). A PCR using ten selected primers was performed using 1.5 µL of this mixture. One γ-^32^P-labeled primer (CG-, CXG-, or CXX-) was used in selective PCR; the other was unlabeled and corresponded to the ends released by the restriction enzyme MseI. The selective PCR product was electrophoresed on 7% polyacrylamide gel. X-ray film was used to visualize the fractionated DNA fragments.

### 2.3. Quantifying Variation Based on the metAFLP Marker System

After separating the selective PCR product on a polyacrylamide gel, a dataset corresponding to DNA cut with Acc65I/MseI and KpnI/MseI enzymes was obtained. The DNA fragments were converted to a binary data set in the form of a zero-one matrix. Visible DNA fragments were coded as ‘1’, while the absence of a DNA fragment was coded as ‘0’. These matrices were then compared. The KpnI/MseI enzyme-derived markers could be used to determine DNA sequence changes (matrix K), whereas markers amplified in the Acc65I/MseI platform (matrix A) reflect sequence and methylation changes. The markers related to DNA methylation changes were evaluated via extraction of the KpnI/MseI markers from those amplified in the Acc65I/MseI (matrix M). The KpnI/MseI-based (matrix K) and that related to DNA methylation (matrix M = A − K) markers were employed to characterize the marker system using GenAlex 6.5 (Excel add-in software) [67]. The percentages of polymorphic loci (%*P*) were evaluated. Furthermore, they were subject to Principal Coordinate Analyses (PCoA) to illustrate relationships between donor plants and their regenerants, representing variation at the DNA sequence and DNA methylation levels.

Moreover, applying the metAFLP approach [64,66], different quantitative characteristics describing sequence variation, DNA de novo methylation, and DNA demethylation regarding symmetric (CG, CHG) and asymmetric (CHH) sequence contexts were evaluated. The evaluation of such characteristics relies on juxtaposing the band patterns evaluated for the donor plant and its regenerants in the Acc65I/MseI and KpnI/MseI platforms. The theoretical background of the respective profiles (for each donor plant and its regenerant), which are coded in four-digit codes, allows their assignment to specific event types. After normalization, the following general quantitative characteristics were evaluated: DNA demethylation (DMV), de novo methylation (DNMV), and sequence variation (SV). The same reasoning could be applied to evaluating quantitative characteristics related to sequence contexts. However, markers amplified by selective primers capable of identifying specific sequence contexts should be used. As a result, SV, DMV, and DNMV regarding CG, CHG, and CHH sequence contexts were evaluated [66]. The quantitative data were used for statistical purposes.

### 2.4. Determination of Glutathione and S-adenosyl-L-methionine Amount in Leaves

Quantification of sulfur metabolites in leaves was performed according to the method proposed by Giustarini and co-workers [68] with slight modifications. The lyophilized leaf samples (5.0 mg) were treated with 1.0 mL of TRIS buffer (pH = 8.0) containing 2 mM of serine and 30 mM of N-ethylmaleimide (NEM) and incubated at 6 °C with shaking. At the end of the incubation, 100 µm of 0.9 M trichloroacetic acid (TCA) was added, and samples were allowed to stand for 10 min. After this time, the samples were centrifuged (13,000 rpm, 10 min). The supernatants were filtered through syringe filters (0.22 µm) and subjected to chromatographic analysis.

The chromatographic separation of 5 μL filtered supernatants was prepared on RP-HPLC-MS/MS Exion LC Triple Quad 5500+ Sciex (SCIEX, Framingham, MA, USA) equipped with Kinetex XB-C18 100A (Phenomenex Ltd., Torance, PA, USA), 100 mm × 2.1 mm, 2.6 µm chromatographic column and Triple Quad 5500+ LC/MS/MS Mass Spectrometer as detector (SCIEX, Framingham, MA, USA). The autosampler chamber and column temperatures were 4 °C and 40 °C, respectively. Eluents were 0.1% solutions of formic acid in water (solvent A) and acetonitrile (solvent B). Elution program at a flow rate of 0.4 mL min^−1^ was carried as follows: 0–0.2 min isocratic elution 100A/0B; 0.2–4 min gradient from 100A/0B to 90A/10B; 4–5 min gradient from 90A/10B to 2A/98B; 5–6 min isocratic elution 2A/98B; 6–7 min gradient from 2A/98B to 100A/0B; 7–9 min isocratic elution 100A/0B to achieve initial analysis conditions. Numbers indicated the percentage amount of eluent A and B. Dwell time 150 msec, collision energy 21V-47V. The identification of chemical compounds was carried out based on MS spectra in positive polarity. Reduced forms of glutathione were determined through the reaction product with NEM to block the sulfhydryl group (GSH-NEM). Identification of the determined compounds was carried out based on molecular fragmentation: for SAM: Multiple Reaction Monitoring (MRM) 1: 399.1 → 250.0; MRM 2: 399.1 → 136.2; for GSSG: MRM1: 613.1 → 355.2; MRM2: 613.1 → 231.1; for GSH-NEM: MRM1: 433.1 → 304.1; MRM2: 433.1 → 201.1. The substances were quantified based on external calibration using a series of solutions with known analyte concentrations.

### 2.5. Determination of the Content of Phytohormones in Leaves

The determination of phytohormones was carried out according to the methodology reported previously [69]. An internal-standard mixture of deuterated IAA, ABA, SA, JA, and ^15^N-zeatin was added to 30 mg of lyophilised, homogenised leaf tissue. Phytohormones were extracted twice with methanol : water : acetic acid (15:4:1, *v*/*v*/*v*) for 30 min at room temperature, followed by 20 min in an ultrasonic bath. After centrifugation, the supernatants were pooled and evaporated to dryness under a gentle stream of N_2_. The residue was re-dissolved in 1 mL 1 M formic acid and centrifuged again (39,000 rpm, 8 min).

Auxins and cytokinins were purified on Oasis MCX solid-phase extraction cartridges (Waters, Milford, MA, USA). After sample loading and a wash with 1 M formic acid, auxins together with JA, SA, and ABA were eluted with 1 mL methanol, whereas cytokinins were eluted with 0.35 M NH_3_ in 60% methanol. Eluates were dried, reconstituted in 100 µL methanol, centrifuged (39,000 rpm, 8 min), and analysed by RP-HPLC-MS.

Chromatography was performed on an Agilent 1260 HPLC coupled to an Agilent 6410B ESI tandem mass spectrometer (Agilent Technologies, Santa Clara, CA, USA) fitted with a Supelco Ascentis RP-Amide column (75 mm × 4.6 mm, 2.7 µm). The injection volume was 7 µL; the eluent flow rate was 0.5 mL min^−1^. Separation and determination of auxins’ fraction were conducted by using solutions of 0.1% formic acid in water (eluent A) and 0.1% formic acid in a mixture of acetonitrile : methanol (1:1; *v*/*v*; eluent B), according to chromatographic program: 0–9 min gradient from 80A/20B to 20A/80B; 9–13 min gradient from 20A/80B to 80A/20B hold by two minutes. ESI settings: positive mode; capillary 4000 V; nebuliser 35 psi; gas temp 300 °C; gas flow 12 L min^−1^. To separate and determine cytokinins’ fraction mobile phases comprised of water + 0.001% acetic acid (eluent A) and acetonitrile (eluent B) and the chromatographic program was used as follows: 0–9 min gradient from 97.5A/2.5B to 90A/10B; 9–13.5 min isocratic elution 90A/10B; 13.5–17 min gradient from 90A/10B to 75A/25B; 17–22 min gradient from 75A/25B to 25A/75B; 22–24 min gradient from 25A/75B to 97.5A/2.5B hold by 2.5 min. ESI settings: positive mode; capillary 3000 V; nebuliser 35 psi; gas temp 350 °C; gas flow 12 L min^−1^. For all analytes, two multiple-reaction-monitoring (MRM) transitions were used for identification and quantification, and ten-point calibration curves were prepared (see Table S1, Supplementary Material).

### 2.6. Determination of Polyamine Amount in Leaves

Estimation of polyamines was performed according to the procedure described earlier [70]. A 5 mg aliquot of lyophilised leaf tissue was extracted twice with 2 × 150 µL 5% HClO_4_ for 30 min at room temperature, followed by 20 min sonication. After centrifugation (39,000 rpm, 8 min), the supernatants were pooled. To 200 µL of this extract were added 10 µL 12% NaOH, 400 µL saturated Na_2_CO_3,_ and 400 µL derivatising reagent (dansyl chloride in acetone, 3.5 g/200 mL). The mixture was kept overnight in the dark at room temperature. Excess dansyl chloride was quenched with proline, and the dansyl-polyamine derivatives were extracted into toluene. The organic phase was evaporated to dryness under N_2_, the residue was dissolved in methanol, centrifuged again (39,000 rpm, 8 min), and subjected to HPLC analysis.

Chromatography was carried out on an Agilent 1260 LC with fluorescence detection and a Zorbax Eclipse XDB-C18 column (75 mm × 4.6 mm, 3.5 µm) thermostatted at 30 °C. The flow rate was 1 mL·min^−1^; mobile phases were prepared by adding 10 mL of glacial acetic acid to 990 mL of water (HPLC purity; eluent A) and mixing methanol with acetonitrile in a volumetric proportion of 3:1 (eluent B). The separation program consisted of the following steps: 0–8.5 min gradient from 45A/55B to 0A/100B; 8.5–9.5 min isocratic elution at 0A/100B; 9.5–10 min gradient from 0A/100B to 45A/55B, followed by a hold for 3.5 min. Detector settings: λ_exc_ = 350 nm; λ_em_ = 510 nm. Polyamines were quantified by external calibration with authentic standards derivatised identically to the samples.

### 2.7. Statistical Analysis

Statistical analyses (Agglomerative Hierarchical Analysis: AHC with Unweighted Pair Group Method with Arithmetic mean: UPGMA as data clustering method with dissimilarity Jaccard coefficients; Principal Coordinate Analysis; Pearson’s correlations analysis; one-way analysis of variance: ANOVA and Ridge Regression Analysis) were performed in XLSTAT 2024.3.0 Excel add-in [71]. PCoA coordinates after Varimax rotation were used to draw 2D plots using R CRAN self-evaluated codes for such purposes. The differences between mean values for metAFLP characteristics (SV, DMV, DNMV) in particular methylation contexts (CHH, CG, CHG) and metabolites (SAM, GSH, GSSG, phytohormones, polyamines) for the regeneration conditions investigated (control vs. tested) were determined using one-way ANOVA (Welsch). The Games–Howell post-hoc test was used to evaluate differences between the tested samples. The Ridge regression analysis, with an alpha parameter set to one, included a single categorical variable as the dependent variable and all quantitative variables from the Common and General models as explanatory variables.

## 3. Results

The final regenerants were obtained from three rye donor plants, D1, D5, and D8, representing double haploid lines (first, fifth, and eighth) derived from the J8 cultivar (Figure 1). The donor plants were the generative progeny of the regenerants derived from the anther cultures. They did not differ in morphology concerning plant growth, leaf shape, colour, width, tillering mode, and number of spikes. Anthers from each donor plant were plated on a control medium (C: C1, C5, C8) and tested media (T: T1, T5, T8). Six separate trials resulted in a total of 137 regenerants. The number of green and albino regenerants in the trials varied from 6 to 140 and 20 to 1314, respectively (Table 1). The highest value of regeneration efficiency expressed as the amount of obtained green regenerants on 100 plated anthers on induction media (green plant regeneration efficiency: GPRE) was observed for donor D8 in the control in vitro condition, but the highest value for regeneration efficiency expressed as the amount of obtained albino regenerants on 100 plated anthers on induction media (albino plant regeneration efficiency: APRE) was observed for donor D5 under Cu-treatment (T5) (Table 1). The doubled haploid regenerants (spontaneously doubled) obtained in all trials exhibited no morphological differences from each other and were in the shape of the donor plant.

### 3.1. MetAFLP-Based Primary Analyses

Depending on the selective primer combination, the KpnI/MseI allowed amplification from 261 to 303 bands (K). In total, 86–168 markers were distinguished utilizing virtual markers related to DNA methylation changes (M matrix). Only one to five unique bands were observed for regenerants obtained under specific in vitro culture conditions related to DNA sequence variation (K matrix). In contrast, the unique band pattern associated with DNA methylation variation showed between 1 and 17 bands (M matrix) (Table 2). The (epi)genetic variation of the regenerants within each treatment is indicated by the percentage of polymorphic loci (*%P*). Loci identified based on the M matrix show higher values than those identified based on the K matrix, indicating higher variation related to DNA methylation and lower variation related to DNA sequence polymorphism (Table 2).

**Figure 2:**
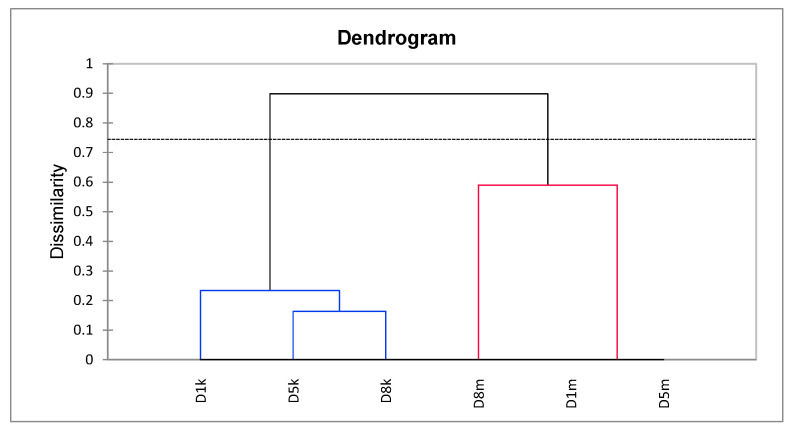
Agglomerative Hierarchical Clustering (UPGMA, Jaccard’s dissimilarity) of representative donor plants (D1, D5, D8), based on sequence (KpnI/MseI; k) and methylation-sensitive (Acc65I/MseI–KpnI/MseI; m) markers. Each donor is represented by two nodes: one reflecting sequence variation (k, blue lines) and one reflecting methylation-sensitive variation (m, red lines). The dendrogram shows two distinct clusters separating samples by marker type (k vs. m), not by donor genotype. The dashed horizontal line indicates the dissimilarity cut-off used to define clusters. The cophenetic correlation coefficient was 0.891, indicating strong consistency between the dendrogram and the original dissimilarity data.

The Agglomerative Hierarchical Clustering (UPGMA, Jaccard coefficient) of D1, D5, and D8 lines based on donor representatives used as sources of explants for further analyses utilizing KpnI/MseI (K) and Acc65I/MseI-KpnI/MseI (M) derived markers showed that the materials were separated into two distinct clusters. However, within each cluster, the differences between representatives were negligible, as indicated by a cut-off line (Figure 2). The cophenetic correlation was 0.891, indicating that the presented relationships were reliable.

### 3.2. Principal Coordinates Analyses Based on the metAFLP Markers

The two binary matrices representing variation at the DNA sequence (K matrix) and DNA methylation (M matrix) levels were subjected to PCoA to explore the relationships between donor plants and their regenerants. The PCoA of sequence variation (metAFLP markers) explained 85% of the variance, and the PCoA based on DNA methylation variation (metAFLP markers) explained 69% of the variance. These analyses offer insight into how genetic and epigenetic changes influence plant regeneration under various treatments. In both cases, Cronbach’s alpha was high (0.998 for sequence variation and 0.993 for DNA methylation), indicating that the results are reliable and reproducible.

The PCoA-based grouping based on sequence and DNA methylation markers resulted in reliable clustering that highlighted differences between donor plants, their regenerants derived under control in vitro anther culture conditions (C1, C5, and C8), and regenerants derived via anther culture with Cu^2+^ (T1, T5) and Zn^2+^ (T8). This grouping reflects the complex interplay between genotype and environmental factors (Cu^2+^ and Zn^2+^) in shaping plant regeneration efficiency.

**Figure 3:**
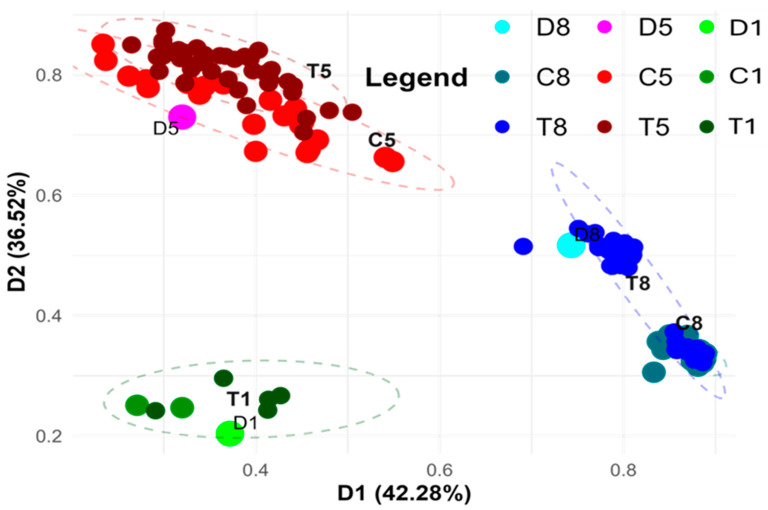
PCoA analysis using the KpnI/MseI platform-based markers (matrix K), illustrating sequence variation between donor (D1, D5, D8) plants and their regenerants. Regenerants represent three lines derived via in vitro anther culture using induction medium without ion supplementation (C1, C5, C8) or supplemented with Cu^2+^ (T1, T5) and Zn^2+^ (T8) ions. The two-dimensional graph visualises the first two principal coordinates (D1 and D2), which together explain 78.8% of the variance. D1 and D2 are statistical axis labels from PCoA and are not donor sample names. Point size reflects sample type: donor samples (D) are the largest, controls (C) intermediate, and treated (T) samples the smallest.

Figure 3 presents the PCoA based on markers generated using the KpnI/MseI metAFLP platform, which captures DNA sequence variation specifically affecting restriction sites and induced by in vitro anther culture treatment. A two-dimensional plot was created to visualise the first two principal components (D1 and D2) after performing a Varimax rotation. The grouping is stable and reliable, supported by high Cronbach’s alpha coefficients of 0.998 and 0.995 for D1 and D2, respectively. This stability suggests that the identified clustering patterns are biologically significant and consistent across multiple data points.

This analysis illustrates the clustering of donor plants representing three lines, along with their regenerants cultured under controlled in vitro conditions and on media supplemented with Cu^2+^ or Zn^2+^ ions. The observed clustering suggests a genotype-specific response to the culture conditions, which is crucial for understanding the role of genetic variation in stress responses during plant regeneration. Regenerants from the first and fifth lines were cultured on media supplemented with Cu^2+^, while those from the eighth line were grown on Zn^2+^-enriched medium.

Samples representing the first line, including two control plants and five Cu^2+^-treated regenerants, cluster closely together in the lower left part of the graph. Notably, no significant differences were observed between regenerants cultured on control versus Cu^2+^-supplemented media in this group. This suggests that Cu^2+^ treatment does not induce significant genetic or epigenetic changes in this particular line, which may indicate that Cu^2+^ exposure does not drastically affect the molecular pathways governing regeneration in this genotype.

In contrast, analysis of the fifth line, which included 21 control and 39 Cu^2+^-treated regenerants, revealed two closely related yet distinct clusters.

The C5 and T5 groups seem to be more spread out in the upper left part of the graph. This suggests that the anther culture caused a lot more variety. The increased variance could mean that this line underwent more genetic or epigenetic reprogramming in response to Cu^2+^ treatment, indicating how the genetic background and the in vitro culture conditions can change each other. Also, the clear distinction between samples from the first and fifth lines implies that there are genetic differences between these lines. But their response to Cu^2+^ seems to be the same, as shown by the fact that they group in the same way under both control and Cu^2+^-treated circumstances.

In contrast, samples from the eighth line, comprising 22 control and 48 Zn^2+^-treated regenerants, form two clearly defined groups. One group includes the control samples along with some Zn^2+^-treated regenerants located in the lower right portion of the graph, while the majority of Zn^2+^-treated samples form a separate cluster. The clear difference between control and Zn^2+^-treated regenerants implies that exposure to Zn^2+^ has a strong effect on the regeneration process, perhaps via changing gene expression or DNA methylation patterns influencing regeneration efficiency. The two clusters in the eighth line are more compact than the more spread-out grouping in the fifth line. This means that they are more stable in current culture conditions and that fewer sequence alterations were likely caused. This could show how the line’s epigenetics change in response to Zn^2+^ treatment, which suggests that the genetic structure is more stable when there is Zn^2+^ stress. Nevertheless, the clear difference between the control and Zn^2+^-treated regenerants suggests that Zn^2+^ ions may have an effect on how plants grow again. This discovery shows that Zn^2+^ has a biological role in changing how well regeneration works, presumably via changing DNA methylation or other epigenetic changes that control gene expression.

Additionally, the separation of eighth-line samples from those of other lines points to potential genotypic effects specific to this line. Thus, the interaction between genotype and Zn^2+^ treatment may underlie distinct epigenetic profiles that influence the success of regeneration.

**Figure 4:**
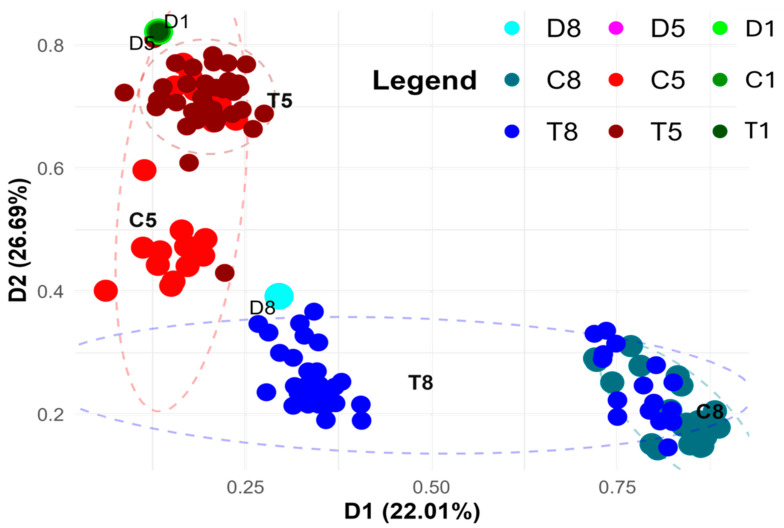
PCoA analysis using the combined Acc65I/MseI and KpnI/MseI platform-based markers (matrix M), illustrating methylation variation between donor (D1, D5, D8) plants and their regenerants. Regenerants represent three lines derived via in vitro anther culture using induction medium without ion supplementation (C1, C5, C8) or supplemented with Cu^2+^ (T1, T5) and Zn^2+^ (T8) ions. The two-dimensional graph displays the first two principal coordinates (D1 and D2), which together explain 56.27% of the variance. D1 and D2 are principal coordinate axes derived from the PCoA analysis and are not donor plant identifiers. Point size reflects sample type: donor samples (D) are the largest, controls (C) intermediate, and treated (T) samples the smallest.

DNA methylation variation among samples derived from three rye lines grown under varying in vitro anther culture conditions, based on virtual methylation markers from the metAFLP platform (Figure 4), reveals that epigenetic differences allow for the grouping of plant materials according to both their genetic origin and specific culture treatments. These epigenetic changes offer insight into how DNA methylation mediates plant responses to stress and affects regeneration efficiency. The grouping is stable, as indicated by Cronbach’s alpha coefficients (0.992 and 0.989 for the D1 and D2 axes). The stability of these groupings suggests that DNA methylation plays a crucial and consistent role in regulating regeneration under various treatment conditions.

The observed clustering is generally consistent with that based on sequence variation (Figure 3). This consistency supports the idea that sequence and epigenetic changes are interrelated and jointly contribute to the efficiency of regeneration. While apparent genotypic (line-specific) effects are evident, such as the distinct clustering of samples from the eighth and fifth lines, marked differences are also apparent between control and treated samples. In the fifth line, some control samples form a separate cluster that includes Cu^2+^-treated regenerants (red dots), whereas the remaining controls are closely grouped with most of the treated samples. This suggests that the epigenetic response to Cu^2+^ treatment in the fifth line is more complex, with some samples showing significant reprogramming, while others remain relatively unaffected.

In contrast, Zn^2+^-treated samples from the eighth line form two distinct subgroups; one includes the treated regenerants and their donor plant, while the other comprises control samples and some treated individuals (lower part of the graph). The appearance of two separate clusters shows that the epigenetic response to Zn^2+^ treatment is different for each one. This could be due to variations in DNA methylation that influence regeneration efficiency. In all cases, the appearance of two separate clusters shows that the treatments had diverse effects on the epigenome.

Interestingly, samples from the first line, both control and Cu^2+^-treated, cluster together with those from the fifth line, regardless of treatment, suggesting that these two lines respond similarly at the DNA methylation level to Cu^2+^ exposure. This means that both lines may have similar ways of regulating their epigenetics when they are exposed to Cu^2+^. These changes could be in DNA methylation that affects how well they regenerate. The groupings, on the other hand, seem to be more spread out than those based on sequence variation. This suggests that epigenetic markers may be more variable than sequence-based markers in these cases.

This suggests that while sequence variation is relatively stable, epigenetic variation in response to Cu^2+^ exposure is more dynamic and potentially more influential in determining the success of regeneration.

### 3.3. MetAFLP Quantitative Characteristics

The metAFLP characteristics and raw biochemical data are provided in Table S2 (Supplementary Materials) and Table S3 (Supplementary Materials). Basic statistics describing sequence variation (SV), de novo methylation (DNMV), and demethylation (DMV) across CHH, CHG, and CG sequence contexts are presented in Table S4 (Supplementary Materials). These markers show that epigenetic reprogramming may be related to the development of microspore embryos. There are other MS-based biochemical indicators, such as S-adenosyl-L-methionine (SAM), glutathione disulfide, reduced glutathione, phytohormones, and polyamines. However, due to sample size limitations, only the metAFLP dataset was fully represented in all treatments.

**Figure 5:**
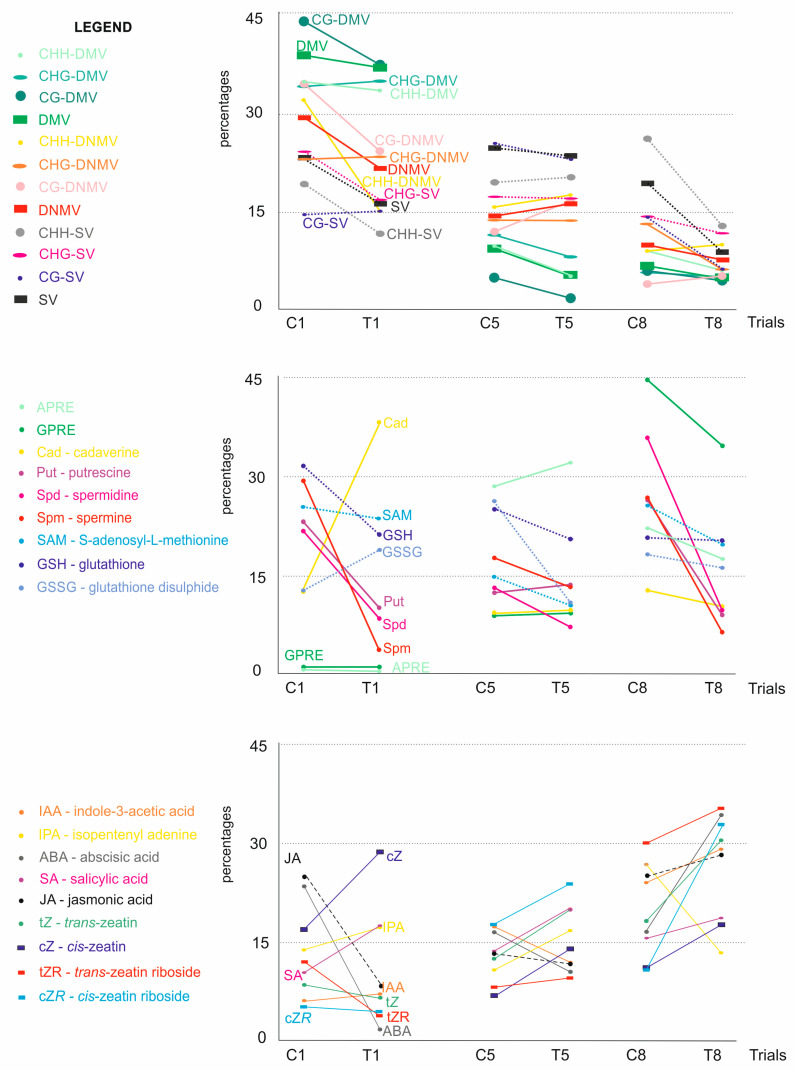
Schematic illustration of the metAFLP characteristics, biochemical marker, and regeneration efficiency of albino and green plants changes due to Cu^2+^ and Zn^2+^ presence in the regeneration medium of anther cultures relative to the control medium without such additives. C1, C5, C8, lines of regenerants obtained on a control medium; T1, T5, T8, lines of regenerants obtained on the tested media T1 and T5 with Cu^2+.^ions, T8 with Zn^2+^ ions. The initial data were normalized and expressed in percentages.

Changes in the metAFLP quantitative characteristics (Figure 5) regarding lines first and fifth treated with Cu^2+^ show that most variables exhibited decreased values compared to the control counterparts. In the first line, represented by only two control and five treated samples, a slight increase in CHG_DMV, CHG_DNMV, and CG_SV was observed. Regarding line fifth, represented by 21 control and 39 treated samples, Cu^2+^ treatment resulted in increased CHH_SV, CHH_DNMV, and CG_DNMV, indicating differences in epigenetic marker changes between the two lines. Analysis of the data generated based on line eighth, which was treated with Zn^2+^, revealed an increase in CHG_DNMV and CG_DNMV, whereas other variables showed decreased values.

### 3.4. Biochemical Characteristics

Regarding polyamines, an increase was evaluated for cadaverine (first line) (Figure 5). Among sulfur-containing metabolites, an increase in GSSG was evidenced due to Cu^2+^ treatment. The fifth line showed increased putrescine and a slight boost in cadaverine in the Cu^2+^ treated trial. The eighth Zn^2+^-treated samples presented a decrease in all polyamines analyzed compared to the controls. Furthermore, all sulfur-containing metabolites were lower in treated samples compared to the control.

Analysis of the growth and stress-related metabolites regarding the first line showed a decrease in JA, ABA, *trans*-zeatin, and *trans*-zeatin riboside (Figure 5). The fifth line revealed increased *cis*-zeatin riboside, *cis*-zeatin, *trans*-zeatin riboside, *trans*-zeatin, IPA, and SA. Zn^2+^ treatment (line eighth) showed a decrease in IPA only.

### 3.5. Plant Regeneration Efficiency

Finally, Cu^2+^ in the in vitro medium failed to change the GPRE (or the change was relatively small) regarding the first line (Figure 5). The same was evidenced for the fifth line. However, an increase in albino was observed for the latter line compared to the former. Regarding line eighth, APRE and GPRE decreased in the treated experiment compared to the control.

**Figure 6:**
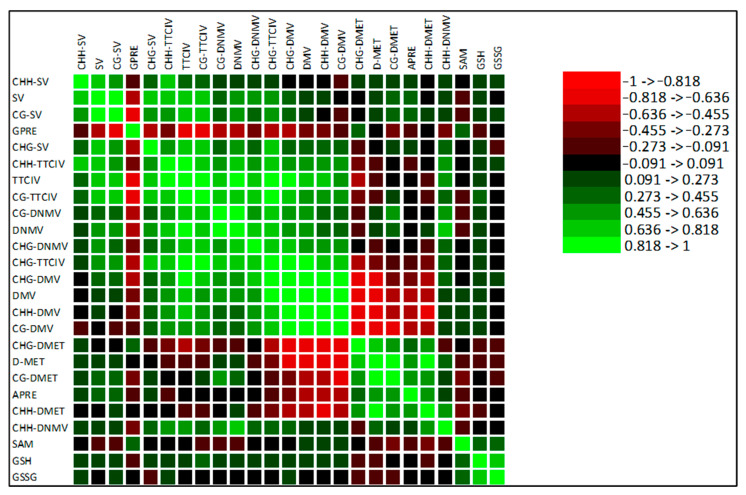
Pearson’s linear correlation heat map for the variables used in the experiment. A row and column represent each variable, and the cells show their correlation. Each cell’s colour represents the correlation’s strength and direction, and darker colours indicate stronger correlations, as shown in the legend, where the values oscillate between −1 and 1.

Due to missing data, only the metAFLP characteristics and some biochemical markers were used for Pearson’s correlation coefficient calculations, showing that the data are primarily uncorrelated or the correlation is moderate (Figure 6). GPRE is significantly and mostly negatively correlated with most of the metAFLP quantitative characteristics, with only a few cases with moderate positive correlations regarding SAM. On the contrary, APRE is positively and negatively correlated with many metAFLP characteristics. Among these, CG_DMV and CHG_DMV show the most substantial negative correlations and appear to be the most determinative in APRE variation, followed by CHH_DMV. A strong positive correlation is also observed for D_MET. Biochemical markers are poorly correlated with the metAFLP variables and moderately or weakly correlated with them.

### 3.6. Analysis of Variance

ANOVA based on 13 metAFLP quantitative characteristics (Table 3) revealed differentiation between three rye lines in control versus Cu^2+^ and Zn^2+^ treated in vitro culture conditions, indicating underlying genotypic differences. Regarding sequence context, lines under control conditions explained from 47% (CG_SV) to 89% (CG_DMV) of the variance in metAFLP characteristics (see *R^2^adj* and *p*-values in Table 3). Games-Howell’s post-hoc test confirmed that demethylation variation (DMV) was present across all sequence contexts and was significantly affected by genotype (line). The most informative dependent variables, with the highest variance explained and statistical significance, were those related to the CG and CHG sequence contexts. In treated samples, the explained variance by sequence context ranged from 37% (CG_DNMV) to 95% (CG_DMV), indicating genotype-dependent epigenetic responses to stress.

GPRE variation was primarily explained by genotype, accounting for 93% of the variance under control conditions and 67% under treatment. On the other hand, the explained variance regarding APRE was minor under control conditions (22%) but significantly higher under stress conditions (71%). This suggests that treatment conditions activate epigenetic processes that facilitate the regeneration of albino and green plants.

The line-specific effects were observed in samples treated with Cu^2+^. In the first line, three metAFLP characteristics significantly differentiated control and treated materials: CG_DMV (*R^2^adj* = 41%), CG_TCIV (26%), and Total_DMV (52%). In all cases, treated samples exhibited higher levels of demethylation variation, indicating Cu^2+^-induced demethylation. These findings are supported by Games–Howell groupings, confirming a measurable epigenetic shift.

Eight metAFLP traits differed significantly between treatments for the fifth line under Cu^2+^ stress. CHH_DMET (*R^2^adj* = 0.55%) showed the most change, indicating that treated plants exhibited more de novo methylation. This finding aligns with the Games–Howell post-hoc data, which revealed a C > T pattern for CHG and CHH sequence context of the metAFLP approach, such as CHG_SV and CHH_DMET. This suggests that treatment lowers demethylation activity (probably due to a stress-induced silencing mechanism).

Line eight, under Zn^2+^ treatment, displayed the most extensive differences, with 16 metAFLP characteristics significantly differentiating treated and control conditions. Although only CHG_DNMV explained a substantial portion of the variance (57%), the Games–Howell comparisons revealed consistent T > C patterns across nearly all sequence contexts (e.g., CG_SV, CG_DMV, CHG_DMV, and CHH_DMET), indicating that Zn^2+^ treatment induced statistically significant increases in demethylation activity in treated materials, suggesting that epigenetically controlled genes involved in regeneration processes are activated possibly due to Zn^2+^-induced demethylation that may facilitate transcriptional reprogramming.

These Games–Howell patterns, which are specific to particular contexts and lines, exhibit various methylation responses. The T > C pattern on line 8 indicates that demethylation is occurring more frequently. This could mean that green plant regeneration is also happening more often. The C > T pattern on line 5 indicates that demethylation is occurring less frequently, which could suggest that regeneration is also happening less often. These results suggest that Zn^2+^ and Cu^2+^ induce distinct epigenetic changes in different genotypes. For example, Zn^2+^ may make chromatin more open, which may help regeneration in some genotypes.

APRE analysis revealed minor changes. In line 5, only 5% of the variance was explained by treatment, with control lines producing slightly more albino regenerants. On the other hand, Zn^2+^ treatment in line 8 explained 16% of the difference in APRE, and this treatment led to more green plant regeneration, which supports the idea that Zn^2+^ causes demethylation. The comparison of the control and treatment samples revealed that lines 1, 5, and 8 exhibited distinct epigenetic responses, reinforcing the finding that epigenetic reprogramming is genotype-specific and more pronounced in lines 5 and 8. Furthermore, control versus treatment comparisons revealed a stronger separation for Zn^2+^-treated samples relative to their controls than for Cu^2+^-treated samples, regardless of genotype, as indicated by both Games–Howell groupings and explained variance patterns.

When treated samples were analysed, polyamines such as spermidine (*p* = 0.02) and spermine (*p* = 0.000) were significantly affected by genotype, explaining the variation in SAM levels (*R^2^adj* = 0.16, *p* = 0.02). Thus, polyamine metabolism may play a role in stress adaptation pathways. The sample grouping corresponded to Cu^2+^ and Zn^2+^ treatments, with the fifth and eighth lines forming distinct groups in the Games–Howell test (Table 4). Again, GPRE remained significantly explained by the line effect under stress (*R^2^adj* = 0.69, *p* = 0.000).

No statistically significant differences (*p* > 0.05) were observed between control and Cu^2+^-treated samples at line 1, suggesting that these materials did not show any significant biochemical responses. On the other hand, Cu^2+^ treatment affected both GSH (*p* = 0.04) and GSSG (*p* = 0.02) in line 5. Thus, oxidative stress may influence the breakdown of glutathione. Additionally, epigenetically controlled genes may be involved in regeneration and are being activated. That is because Zn^2+^-induced demethylation may facilitate transcriptional reprogramming.

These Games–Howell post-hoc tests demonstrate different methylation changes found in specific contexts and lines. The T > C pattern on line 8 indicates that demethylation dominates in treated materials, which is consistent with GPRE. The C > T pattern on line 5 shows that demethylation is less pronounced in treated materials. Thus, Zn^2+^ and Cu^2+^ affect the epigenome in distinct ways in different genotypes, which corresponds with the observed stress-induced methylation shifts in this line.

The comparison between the control and Zn^2+^-treated samples in line 8 showed significant changes in putrescine (*p* = 0.04), spermidine (*p* = 0.02), and spermine (*p* = 0.01). It means that Zn^2+^ has a significant effect on how polyamines are broken down.

Most notably, GPRE was significantly increased in Zn^2+^-treated samples (*p* = 0.000), highlighting the biologically significant link between polyamine-based epigenetic regulation and green plant regeneration.

Although APRE responses in line 5 were less pronounced, the variance explained by treatment was minimal (*R^2^adj* = 0.05, *p* = 0.03), with slightly more albino regenerants observed under control conditions. It was observed that in line 8, Zn^2+^ treatment significantly increased GPRE (*p* = 0.000), indicating that Zn^2+^ may promote more efficient regeneration of green plants than albino ones through epigenetic processes.

These results indicate that biologically important markers, particularly polyamines (Spd, Spm) and antioxidants (GSH, GSSG), are consistent with changes in GPRE, supporting the concept that differences in regeneration efficiency may be due to genotype-dependent biochemical responses to stress.

### 3.7. Ridge Regression Analysis

Ridge regression analysis (Table 5) allowed the identification of key molecular and biochemical predictors of regeneration capacity (GPRE and APRE). For GPRE, several metAFLP sequence context characteristics were consistently included across the Common and General models. CHH_SV was the only characteristic positively associated with GPRE. This may suggest that the CHH context may be involved in green plant regeneration. On the other hand, the adverse effects of CG_SV, CHG_SV, CHG_DMV, and CG_DNMV imply that increased stability or demethylation within CG and CHG contexts is associated with reduced regenerative potential.

Among biochemical traits, the most positive effect on GPRE was seen for S-adenosyl-L-methionine across all models (e.g., coefficient = 377.73 in the Common model; 331.27 in the Extended General model). Again, such a result reinforces the role of methyl donor availability in promoting regeneration. Moreover, putrescine also exhibited a slight favourable effect. The result aligns with its role in cell growth and morphogenesis.

The Ridge regression results for APRE showed that CHH_DNMV and CG_SV were positive contributors. This suggests that de novo methylation and CG context methylation may be related to the growth of albino plants. On the other hand, CHH_SV and CG_DMV had an adverse effect on APRE. Interestingly, SAM is again a significant positive predictor of APRE (especially in the extended General model, coefficient = 1922.24). The presented data suggest that SAM plays a central role in epigenetic control. Cadaverine also contributed positively to APRE (e.g., coefficient = 798.43), potentially reflecting stress-induced modulation of albino plant development. Other variables, including most phytohormones, displayed minimal or inconsistent effects across models, suggesting limited or context-specific roles in regeneration.

Taken together, these findings suggest that CHH-associated methylation flexibility and SAM-mediated methyl donor availability are biologically essential regulators of both albino and green plant regeneration efficiency. Ridge regression thus supports the hypothesis that genotype-dependent epigenetic and metabolic adjustments are central to in vitro regenerative capacity.

### 3.8. Analysis of Amino Acid Sequence Related to Sulfur-Containing Metabolites and Polyamines Requiring Metal Ions for Their Activity

**Figure 7:**
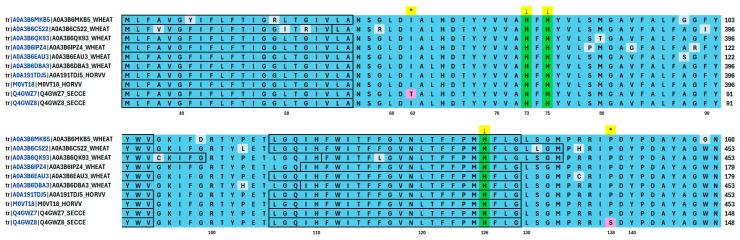
Partial alignment of cytochrome *c* oxidase subunit I AAs sequence within the expected active center for rye, barley, and wheat. Helical, transmembrane sequences are marked in frames. Arrows and green colour indicate the histidines involved in the active centre, * show differences in AAs across genotypes.

Analysis of amino acid (AA) sequences available in the UniProt database (Source: https://www.uniprot.org/ accessed on 25 March 2025) showed that only those for the cytochrome *c* oxidase subunit I are available for rye (Q4GWZ8, Q4GWZ7), barley (A0A191TDJ5, M0VT18), and wheat (A0A3B6MKB5, A0A3B6C522, A0A3B6QK93, A0A3B6IPZ4, A0A3B6EAU3, A0A3B6DBA3) simultaneously. The rye AA sequence, which is 219 AA in length, fits the barley sequence, starting from 306 and ending at 524 amino acids. Similarly, wheat sequence and rye are aligned starting from 13 and ending at 231 (A0A3B6MKB5), from 32 to 250 (A0A3B6IPZ4, A0A3B6EAU3), from 306 to 524 (A0A3B6QK93, A0A3B6DBA3), and from 306 to 515 (A0A3B6C522) of wheat AAs (Figure 7).

A comparison of rye AA sequences identified two AAs near the active centre (represented by conserved histidine at 73, 75, and 126 positions of the rye sequence) of the cytochrome *c* oxidase subunit I. The difference is due to the 62nd position of rye AA, where threonine and isoleucine reside. The second difference is at the 138 position, where serine and proline reside. Interestingly, the respective AAs are always represented by isoleucine and proline in wheat and barley, suggesting their conserved nature in other cereals.

**Figure 8:**
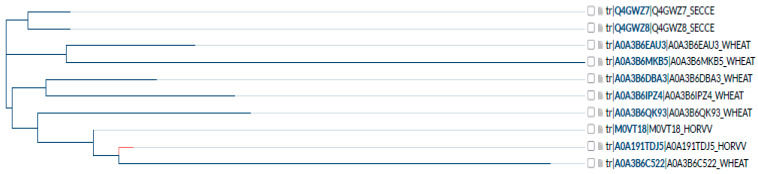
Dendrogram illustrating relationships (similarity) between cytochrome *c* oxidase subunit I amino acid sequences available for two rye (Q4GWZ8, Q4GWZ7), two barley (A0A191TDJ5, M0VT18), and six wheat (A0A3B6MKB5, A0A3B6C522, A0A3B6QK93, A0A3B6IPZ4, A0A3B6EAU3, A0A3B6DBA3) AA sequences.

Nevertheless, other AA differences of the cytochrome *c* oxidase subunit I were found, which are reflected in the dendrogram (Figure 8). They do not affect grouping according to species and are not involved in the enzyme’s active centre being randomly distributed across analyzed sequences.

## 4. Discussion

The cultivar J8 was used in rye breeding programs and was subject to several selfing cycles that made it morphologically uniform, but its uniformity was not tested at the DNA level. Randomly chosen plants of the cultivar were employed to derive several sublines via in vitro anther cultures. Three lines, the outcome of subsequent cycles encompassing in vitro anther cultures and selfing regenerants, exhibited the highest androgenic ability. Representatives of each line were used to have progeny utilized as donors for subsequent experiments. Following the way the materials were evaluated, the lines’ donor plants were considered highly related, except that they could have represented a narrowed part of the cv J8 available variation and might also differ due to a well-described phenomenon called somaclonal variation [72] induced during in vitro anther tissue culture.

Nevertheless, the lines’ donor plants preserved high androgenic ability and morphological identity. Besides, representatives of lines’ donor plants used as sources of explants were (epi)genetically identical (negligible differences were evaluated via UPGMA analysis), also supporting their high (epi)genetic similarity. Thus, we have assumed the comparison of experimental trials regarding different treatments of anthers with Cu^2+^ and Zn^2+^ vs. controls and comparing results based on lines’ donor plant regenerants using (epi)genetic and biochemical markers as justified and the only reasonable option to derive sets encompassing numerous regenerants required for statistical purposes. The issue’s significance is reflected by line 1 donor plant regenerants, where the number of regenerants evaluated under control and treated conditions was low, resulting in biased outcomes regarding (epi)genetic and biochemical levels.

As can be seen, copper ions increase (line 1 donor plant regenerants) and decrease (line 5 donor plant regenerants) the total number of green plants. Zn^2+^ also decreases the count regarding treated vs. control trials. While the results presented for line 1 donor plant regenerants should be treated with caution due to low representation of regenerants, the other trials were numerous and should be considered reliable. GPRE and APRE reflect this regarding lines 5 and 8 donor plant regenerants, where a reversed relationships were observed.

Based on our experiments, it is not apparent whether the adverse action of the ions in rye anther culture regarding GPRE results from inadequate concentration of the ions in the IM, as their optimization was not the study’s goal. However, the option cannot be ruled out. Our earlier studies in triticale and barley have demonstrated (at least for Cu^2+^) that optimized concentration may increase green plant regeneration efficiency [14,30]. However, the adverse effect of zinc is congruent with data presented by others where zinc oxide nanoparticles used during micropropagation of stevia negatively influenced shoot length, root number and length, and the fresh weight of the plantlets [73]. Also, using a higher-than-optimal Zn^2+^ concentration during micropropagation of *Rauvolfia serpentina* negatively affected the morphogenic potential [74]. In contrast, microspore embryogenesis had a differential efficiency concerning the supplementation of induction media with copper sulphate in various wheat genotypes [75]. The presented results suggest species-specific and genotype-specific reactions. Thus, understanding differences may impact plant tissue culture in cereals.

Furthermore, PCoA results suggest the presence of line donor plants and their regenerants, with differences in regeneration. Utilizing DNA sequence markers, the eighth-line donor plant differed from the first and the fifth-line donor plant (and their regenerants). The latter two donors also exhibited dissimilarities. Furthermore, control and Zn^2+^ regenerants formed separate groups. However, the distinctiveness between controls and Cu^2+^ treated samples was less pronounced. Interestingly, the regenerants of the fifth-line donor plant were dispersed compared to those of the eighth-line donor plant. It may suggest that Cu^2+^ treatment induces more mutations than Zn^2+^, possibly because Cu^2+^ may participate in Fenton’s Haber–Weiss reaction, inducing ROS production [76]. The latter may be responsible for mutations [77]. However, the respective controlled condition-derived regenerants also show apparent dispersion on the PCoA, suggesting that the fifth-line donor regenerants may be susceptible to mutations under in vitro tissue culture conditions. Interestingly, most of the metAFLP quantitative characteristics related to sequence contexts dropped in Zn^2+^ samples, whereas in the Cu^2+^ treated (fifth-line donor plant-derived regenerants), the decrease and increase were evidenced. Besides, ANOVA demonstrated the significance of DNA sequence differences regarding all analyzed regenerants but with some variation concerning line donor plant-derived regenerants regarding sequence contexts. On the other hand, Cu^2+^ is a component of Cu/Zn SOD [5]; it may participate in ROS scavenging [78]. Thus, a delicate balance in Cu^2+^ ion concentration is needed not to induce mutations.

It is worth mentioning that utilizing markers related to methylation changes the PCoA grouping of line donor plants and their regenerants derived on Cu^2+^/Zn^2+^ containing IM, which is even more noticeable than in the case of DNA sequence-related markers. The observed grouping may reflect epigenetic changes affecting the nuclear genome during reprogramming from gametophytic to sporophytic fate [79] or somaclonal variation combined with the action of used metal ions. ANOVA confirmed the significance of DNA methylation changes in all trials. The presented data are congruent with our earlier studies on barley [30] and triticale [31], where methylation changes were involved in explaining GPRE. Thus, epigenetic aspects of microspore reprogramming are essential for a complete image of processes running in anther cultures.

Copper and zinc are involved differently in DNA methylation and plant regeneration processes. Copper, as a cofactor in enzymes such as Cu/Zn superoxide dismutase (SOD) [5] and cytochrome *c* oxidase [16], may impact reactive oxygen species (ROS) homeostasis [78] and energy metabolism, thereby affecting methylation through oxidative stress modulation [80]. Copper’s dual role in ROS production and scavenging could explain the mixed pattern of mutations and methylation changes observed. Nevertheless, zinc’s involvement in epigenetic processes is important because many enzymes involved in epigenetic regulation require this metal. Among these, zinc-coordinating proteins, which modulate epigenetic processes such as DNA methylation, histone modifications, and the expression of non-coding RNA, are extremely important [81]. Copper and zinc can affect plant regeneration in different ways. CuSO_4_ concentrations above the standard dosage in the medium improved the organogenesis of cucumber adventitious shoots [82]. Additionally, they had a positive effect on obtaining different cereal species through in vitro cultures, as demonstrated by triticale [22], barley [83], indica rice [27], and sorghum [26]. But negative effects of overexposure to this micronutrient in tetraploid wheat have also been reported [23]. However, elevated Zn^2+^ levels have been reported to impair morphogenic potential, which is consistent with the negative effects of zinc observed in our experiments. Thus, copper’s effect on DNA methylation and plant regeneration may be mediated via its effect on ROS balance and enzymatic cofactors. Zinc, on the other hand, is most likely involved in structural and regulatory aspects that impact epigenetic enzyme activity and metabolite pathways. These findings are consistent with observations from other cereal species demonstrating genotype-specific responses to Cu and Zn in microspore embryogenesis and tissue culture.

One of our hypotheses was that metal ions participating as cofactors of enzymatic reactions may modify biochemical pathways. Such modification may influence APRE and GPRE. Studies on triticale [84] and barley [30] regarding anther culture plant regeneration showed that Cu^2+^ ions in the induction medium affected S-adenosyl-L-methionine synthesis and impacted glutathione. The data presented were interpreted in terms of changes affecting the electron transport chain, explicitly pointing to the copper ion-dependent complex IV (cytochrome *c* oxidase: COX) [16], the Yang cycle-producing SAM [85,86], and the transsulfuration pathway, which produces, among others, glutathione [87]. Thus, it became apparent that sulfur-containing metabolites and those associated with polyamine synthesis and requiring metal ion cofactors could influence APRE and GPRE.

The notion was partly confirmed by analyzing changes in metabolites revealed between control regenerants derived without Cu^2+^ or Zn^2+^ ions in the IM and those regenerated in their presence. Most of the metabolites we have analyzed underwent a decrease in treated samples compared to the controls. The changes affected sulfur-containing metabolites and those related to growth and stress (polyamines and phytohormones). Regenerants derived from Cu^2+^-containing medium underwent less dramatic changes than those with Zn^2+^. It should be noted that the experiment conducted with the first line donor plant-derived regenerants was limited to seven plants. Thus, the conclusions concerning those materials should be cautious. All the changes regarding Cu^2+^ treatment failed to improve (or improvement was not apparent) GPRE; however, the APRE increased (fifth-line donor plant-derived regenerants). It may suggest that the PSII–PSI chloroplast electron transport is affected.

The PSII to PSI system that functions in chlorophyll-containing cells but also in plastids consists of plastoquinol, cytochrome *b_6_-f*, and plastocyanin as electron carriers. The cytochrome *b_6_-f* complex, which contains hemes and the ‘Rieske’ iron–sulfur protein (2Fe-2S) [88], is responsible for cyclic electron transport. This process depends on the photochemical reactions of PSII and passes through the cytochrome *b_6_-f* complex [89]. The activity of this complex can be blocked by the presence of Cu^2+^ and Zn^2+^ ions, which act similarly and inhibit plastoquinol (PQH_2_) binding by restraining the hydrophilic head domain of the ‘Rieske’ iron−sulfur protein [90]; thus the chloroplast linear electron transfer and/or cyclic electron transfer [91] proper functioning may be somehow influenced. Furthermore, plastocyanin requires Cu^2+^ for its functioning. Thus, the PSII–PSI system may be sensitive to metal ions in the IM. The presented data are in line with our results.

It was demonstrated that Cu^2+^-treatment (see results related to the regenerants of the fifth line) resulted in an APRE increase, whereas GPRE was nearly at the same level. The Zn^2+^ ions mainly negatively affected all metabolites analyzed. However, an apparent drop in polyamine levels was observed. Moreover, a decrease in APRE and GPRE was also evidenced, confirming our results regarding the adverse action of the ion on regeneration efficiency. Advanced studies on the PSII-PSI system involving cytochrome *b_6_-f* and plastocyanin protein sequence may help increase GPRE in rye and other cereals. Nevertheless, the current study was conducted on a few lines, and ion concentrations in the IM were not optimized and could be suboptimal, affecting results.

Interestingly, phytohormones also changed due to Cu^2+^ and Zn^2+^ treatment, showing that the ions produced a stressful environment, as, in nearly all cases, an increase in their levels was observed. The presented results are congruent with the notion that under stressful conditions, an increase in phytohormone levels is expected [92,93]. Under such conditions, most probably not optimal for GPRE, it is apparent why GPRE was lower than under control conditions. Most of the changes in metabolites were confirmed by ANOVA, and they were well documented for the fifth line donor plant-derived regenerants, especially for the eighth one. It became apparent that identifying the most significant factors (metAFLP and metabolites) requires implementing sophisticated statistical tools based on regression analysis.

As Ridge regression analyses indicate, sulfur-containing metabolites (SAM) and polyamines (cadaverine) are the most essential compounds affecting APRE and GPRE. Changes regarding Cu^2+^ and Zn^2+^ treatment affecting sulfur-containing metabolites, polyamines, and phytohormones impacting GPRE and APRE may reflect problems with functioning enzymes involved in respective biochemical pathways and depending on metal ions. In the case of SAM synthesis, a possible candidate enzyme that requires Cu^2+^ and Fe^2+^/Fe^3+^ metal cofactors is a complex IV–cytochrome *c* oxidase [94], which is a part of the electron transport chain [95] and is involved in oxygen reduction [96]. The cytochrome *c* oxidase amino acid sequence analysis showed that only rye, wheat, and barley sequences were available for the cytochrome *c* oxidase subunit I. The alignment of the sequences showed that the position of histidines forming the active centre was conserved and that in the very vicinity of the centre, in the case of one of two rye sequences, proline (P) changed to serine (S) and threonine (T) changed to isoleucine (I). Although the changes may be random, it cannot be excluded that they may also reflect the variable androgenic ability of rye genotypes. If this is the case, the enzyme might be a candidate for genome editing procedures in future experiments. Further experiments, including rye genotypes exhibiting varying androgenic ability, are needed to confirm the significance of P to S (or others) change for androgenic ability in rye.

Regarding polyamine synthesis, ornithine decarboxylase (ODC) that catalyzes putrescine synthesis [97,98] and other enzymes representing an alternative pathway of polyamine synthesis such as arginine decarboxylase-1 (ADC1) synthesizes agmatine [99] and agmatine iminohydrolase (AIH), also known as agmatine deiminase, converts agmatine to N-carbamoylputrescine [100] depend on pyridoxal phosphate (PLP) cofactor that does not need metal ion cofactors for functioning. The enzymes are not candidates for future manipulations to improve GPRE via anther culture in rye.

Copper Amine Oxidase is another enzyme that influences polyamine catabolism by breaking down, i.e., putrescine [34,101]. It is responsible for 4-aminobutyraldehyde synthesis from putrescine. The enzyme is indirectly linked to plant cadaverine biosynthesis via its catabolism [102]. It should be noted that increased cadaverine levels increased GPRE (T5 with Cu^2+^). CuAOx contains copper atoms in its active site, which are crucial for the oxidative deamination of amines. The copper ions participate in electron transfer during the catalytic cycle, enabling the conversion of amines into aldehydes, ammonia, and hydrogen peroxide. From this point of view, it is unsurprising to see that the presence of Cu^2+^ in the induction medium increases the cadaverine level.

On the other hand, cadaverine was reduced in the presence of Zn^2+^, suggesting that zinc may compete for the enzyme’s active centre, affecting its functioning. Considering its significant effect on APRE and less pronounced one on GPRE (as indicated by regression analysis), it is worth considering the enzyme as a putative target for genetic manipulation directed towards GPRE increase and APRE decrease. Unfortunately, a search of the protein database (UniProt) failed to identify respective sequences regarding barley, wheat, rye, or other cereals.

## 5. Conclusions

The presented study illustrates that anther culture plant regeneration involves multilevel changes in biochemical metabolites and DNA sequence and methylation patterns that might be responsible for rye GPRE and APRE. It was demonstrated that among polyamines, cadaverine was the most influential on APRE and, to a lesser extent, on GPRE, whereas SAM affected GPRE the most. While the other metabolites or DNA methylation changes might have some impact on APRE and GPRE, their effects were less pronounced. The study’s results could be implemented in tissue culture experiments involving SAM and cadaverine as the IM additives to verify whether their presence helps improve GPRE in rye. Our study has also demonstrated that the presented approach may be used to identify putative candidates for gene editing procedures that may impact plant regeneration efficiency in rye. Hence, in-depth studies are needed to test, at least, the effect of exogenously applied cadaverine on the efficiency of the androgenic response in different rye genotypes or other cereal species in general. The main limitations of the study are the small sample size used, the lack of biological replicates, and the limited number of genotypes investigated. However, androgenic genotypes, especially those unrelated, are rare, which limits the opportunity to extend the study and its generalisation. Thus, the presented data regarding the role of Cu^2+^ and Zn^2+^ ions should be treated as preliminary results that require further investigation.

## Figures and Tables

**Table 1:** Number of albino and green regenerants obtained under control and modified in vitro anther culture conditions with varying Cu^2+^ and Zn^2+^ concentrations and regeneration efficiency.

Trial	In Vitro Anther Culture Conditions		Total Number of Regenerants		Regeneration Efficiency ^1^		Number of Green Regenerants Employed in metAFLP
	Cu (μM)	Zn (μM)	Albino	Green	APRE	GPRE	
C1	-	-	40	6	12.90	1.94	2
T1	5	-	20	13	5.97	3.88	5
C5	-	-	1207	78	482.8	31.2	21
T5	5	-	1314	63	503.45	24.14	39
C8	-	-	371	140	403.26	142.17	22
T8	-	150	392	133	296.27	99.25	48

^1^ Regeneration efficiency, number of regenerants obtained per 100 plated anthers; APRE, albino plant regeneration efficiency; GPRE, green plant regeneration efficiency; C1, C5, C8, lines of regenerants obtained on a control medium; T1, T5, T8, lines of regenerants obtained on the tested media T1 and T5 with Cu^2+.^ions, T8 with Zn^2+^ ions.

**Table 2:** Total band patterns data for regenerants obtained in different trials for sequence (K) and methylation (M) pattern change markers.

Band Characteristics	Matrix ^1^	Trial					
		C1	T1	C5	T5	C8	T8
No. bands	K	261	274	303	301	273	281
	M	86	80	164	168	91	127
No. private bands	K	1	0	5	2	0	0
	M	7	4	9	17	1	3
%*P*	K	4.26	9.66	23.86	19.89	5.97	14.77
	M	9.66	10.80	40.91	41.76	17.33	29.83

^1^ K, sequence data (K-matrix); M, methylation data (M-matrix); No. Bands, number of different bands; No. Private Bands, number of bands unique to a single population; %*P,* percentage of polymorphic loci; C1, C5, C8, lines of regenerants obtained on a control medium; T1, T5, T8, lines of regenerants obtained on the tested media T1 and T5 with Cu^2+.^ions, T8 with Zn^2+^ ions.

**Table 3:** The arrangement of ANOVA results reflects how lines used affect metAFLP characteristics and plant regeneration efficiency under in vitro anther tissue cultures of rye and demonstrates sources of variations between regenerants grown under control and modified conditions within and between lines. Albino and green plant regeneration efficiency is also included.

			metAFLP Quantitative Characteristics																				Regeneration	
			Sequence Contexts															Total						
Samples	Statistics	Line	CHH					CHG					CG											
			SV *	DMV	DNMV	TCIV	D-MET	SV	DMV	DNMV	TCIV	D-MET	SV	DMV	DNMV	TCIV	D_MET	SV	DMV	DNMV	TCIV	D_MET	APRE	GPRE
Controls	*R^2^_adj_*		0.22	0.72	0.31	0.35	0.16	0.39	0.83	0.14	0.64	0.66	0.47	0.89	0.56	0.56	0.49	0.13	0.85	0.57	0.57	0.50	0.22	0.93
	*F(Welch)*		8.42	259	5.48	5.05	3.17	9.46	161	8.67	64.52	29.75	15.7	3983	36.93	471.4	20.25	4.42	4049	13.17	41.01	7.20	1.54	567.7
	*p*		0.07	0.00	0.11	0.13	0.19	0.06	0.00	0.06	0.00	0.02	0.02	0.00	0.01	0.00	0.02	0.14	0.00	0.04	0.01	0.09	0.36	0.00
	Games- Howell	1		A					C		C	A	A	B	B	C	A		C	B	B			A
		5		A					B		B	A	B	A	B	B	A		B	B	B			B
		8		B					A		A	A	A	A	A	A	A		A	A	A			C
Treated	*R^2^_adj_*		0.19	0.91	0.13	0.32	0.48	0.37	0.94	0.56	0.80	0.66	0.72	0.95	0.37	0.72	0.54	0.56	0.98	0.41	0.71	0.67	0.71	0.69
	*F(Welch)*		14.5	216	8.32	33.07	151.59	25.2	458	96.95	273.5	52.09	132	207	42.70	118.85	124.41	62.1	2776	61.37	176.62	237.59	689.1	222.2
	*p*		0.00	0.00	0.00	0.00	0.00	0.00	0.00	0.00	0.00	0.00	0.00	0.00	0.00	0.00	0.00	0.00	0.00	0.00	0.00	0.00	0.00	0.00
	Games- Howell	1	A	B	AB	C	A	AB	C	A	A	A	B	C	B	C	A	B	B	C	C	A	A	A
		5	B	A	B	B	C	B	B	B	B	C	C	A	B	B	C	C	A	B	B	C	C	B
		8	A	A	A	A	B	A	A	C	C	B	A	B	A	A	B	A	A	A	A	B	B	C
Controls vs. Treated	*R^2^_adj_*		0.51	−0.11	0.59	0.52	0.55	0.43	−0.14	−0.19	0.40	−0.19	−0.19	0.41	0.47	0.26	−0.02	0.57	0.52	0.45	0.59	0.25	0.08	−0.19
	*F(Welch)*		7.48	0.78	3.30	4.09	3.44	5.41	0.26	0.03	3.95	0.03	0.06	14.3	6.60	7.71	0.61	8.37	15.7	2.73	6.33	1.23	0.00	0.15
	*p*		0.11	0.42	0.31	0.25	0.30	0.16	0.66	0.89	0.22	0.88	0.82	0.02	0.13	0.04	0.54	0.12	0.01	0.32	0.18	0.45	1.00	0.72
	Games–Howell	1												T > C		T > C			T > C					
Controls vs. Treated	*R^2^_adj_*		−0.01	0.33	−0.01	−0.01	0.09	−0.02	0.30	−0.02	0.04	0.11	0.03	0.23	0.03	0.00	0.12	−0.01	0.37	0.00	0.01	0.17	0.05	−0.02
	*F(Welch)*		0.13	20.8	0.49	0.58	7.82	0.07	17.1	0.00	2.69	8.24	1.68	12.6	3.72	0.52	11.29	0.32	21.2	1.38	1.21	16.04	4.89	0.14
	*p*		0.72	0.00	0.49	0.45	0.01	0.79	0.00	0.97	0.11	0.01	0.21	0.00	0.06	0.48	0.00	0.58	0.00	0.25	0.28	0.00	0.03	0.71
	Games–Howell	5		T > C			C > T		T > C			C > T		T > C			C > T		T > C			C > T	C > T	
Controls vs. Treated	*R^2^_adj_*		0.44	0.35	−0.01	0.37	0.08	0.17	0.02	0.57	0.33	0.59	0.31	0.13	0.00	0.22	0.05	0.37	0.32	0.09	0.36	−0.01	0.05	0.16
	*F(Welch)*		116	37.9	0.29	72.68	6.25	19.2	2.93	81.13	39.79	95.95	64.7	11.7	1.37	40.72	6.51	86.4	33.9	9.84	77.77	0.05	2.34	21.38
	*p*		0.00	0.00	0.59	0.00	0.02	0.00	0.09	0.00	0.00	0.00	0.00	0.00	0.25	0.00	0.01	0.00	0.00	0.00	0.00	0.82	0.14	0.00
	Games–Howell	8	T > C	T > C		T > C	C > T	T > C		T > C	T > C	T > C	T > C	T > C		T > C	C > T	T > C	T > C	T > C	T > C			T > C

* SV, sequence variation; DMV, demethylation; DNMV, de novo methylation; D-MET, change in methylation; CHH, CHG, CG sequence contexts; 1, 5, 8, analyzed lines; Regeneration efficiency, regenerants obtained per 100 plated anthers; APRE, albino plant regeneration efficiency; GPRE, green plant regeneration efficiency; C-control conditions; T, treated conditions; T > C means that T and C form separate groups based on post-hoc test; A, B, and C reflect post-hoc groups. Interestingly, similar analyses conducted on biochemical markers for control samples failed to be explained by lines (Table 4, all *p* > 0.05, except for GPRE, *p* = 0.000), indicating that in non-stressed conditions, genotype has a limited influence on biochemical variation. Only GPRE was significantly explained by the line effect (*R^2^adj* = 0.93, *p* = 0.000), confirming that green plant regeneration is strongly genotype-dependent under control conditions.

**Table 4:** The arrangement of ANOVA results reflects how lines used affect biochemical variables and plant regeneration efficiency under in vitro anther tissue cultures of rye, demonstrating sources of variation between regenerants grown under control and modified conditions within and between lines. Green plant regeneration efficiency is also included.

Sample	Statistics	Line	Biochemical Variables/Growth and Stress-Related Metabolites																**Regeneration**	
			Sulfur-Containing Metabolites			Polyamines				Phytohormones										
			SAM *	GSH	GSSG	Put	Cad	Spd	Spm	IAA	IPA	ABA	SA	JA	tZ	cZ	tZR	cZR	**APRE**	**GPRE**
Controls	*R^2^_adj_*		0.14	0.13	0.02	0.01	0.02	0.05	−0.02	0.04	-0.01	−0.04	−0.02	0.25	0.08	0.12	0.05	−0.02	0.22	0.93
	*F(Welch)*		3.71	2.55	0.99	0.97	1.11	1.73	0.46	0.67	0.62	0.00	0.23	5.34	1.61	1.74	1.66	0.31	1.54	567.70
	*p*		0.16	0.24	0.47	0.48	0.45	0.33	0.67	0.58	0.60	1.00	0.81	0.12	0.35	0.33	0.35	0.76	0.36	0.00
	Games–Howell	1																		A
		5																		B
		8																		C
Treated	*R^2^_adj_*		0.16	−0.02	0.03	−0.02	0.26	0.08	0.33	0.01	−0.13	0.15	−0.13	0.17	0.04	−0.09	0.16	−0.08	0.71	0.69
	*F(Welch)*		6.89	0.22	1.61	0.20	0.90	12.87	17.27	1.09	0.07	2.45	0.09	2.67	1.35	0.31	2.50	0.41	689.14	222.25
	*p*		0.02	0.81	0.26	0.83	0.46	0.02	0.00	0.36	0.94	0.12	0.91	0.10	0.29	0.74	0.12	0.67	0.00	0.00
	Games–Howell	1	B					AB	A										A	A
		5	A					A	B										C	B
		8	B					B	A										B	C
Controls vs. Treated	*R^2^_adj_*		−0.25	0.25	−0.20	0.14	−0.23	0.79	0.97	NA	NA	NA	NA	NA	NA	NA	NA	NA	0.08	−0.19
	*F(Welch)*		0.02	1.24	0.19	1.50	0.43	11.98	97.77	NA	NA	NA	NA	NA	NA	NA	NA	NA	0.00	0.15
	*p*		0.90	0.45	0.70	0.35	0.58	0.07	0.01	NA	NA	NA	NA	NA	NA	NA	NA	NA	1.00	0.72
	Games–Howell	1							NA											
Controls vs. Treated	*R^2^_adj_*		0.04	0.13	0.16	−0.02	−0.02	0.02	0.01	−0.03	−0.03	−0.01	0.04	−0.07	0.07	0.03	−0.07	−0.07	0.05	−0.02
	*F(Welch)*		2.18	4.79	6.82	0.02	0.18	1.08	0.85	0.59	0.48	0.88	1.52	0.05	1.90	1.27	0.10	0.14	4.89	0.14
	*p*		0.09	0.04	0.02	0.90	0.67	0.31	0.37	0.46	0.51	0.37	0.24	0.82	0.20	0.29	0.77	0.72	0.03	0.71
	Games–Howell	5		T > C	T > C														C > T	
Controls vs. Treated	*R^2^_adj_*		0.03	−0.01	−0.02	0.12	0.01	0.17	0.18	−0.05	−0.03	0.08	0.00	−0.04	0.10	0.00	−0.05	0.13	0.05	0.16
	*F(Welch)*		2.45	0.43	0.30	4.87	1.45	6.86	7.33	0.18	0.46	2.46	1.06	0.32	2.74	0.86	0.08	3.30	2.34	21.38
	*p*		0.13	0.52	0.59	0.04	0.24	0.02	0.01	0.68	0.51	0.14	0.32	0.59	0.12	0.37	0.78	0.10	0.14	0.00
	Games–Howell	8				T > C		T > C	T > C											T > C

* SAM, S-adenosyl-L-methionine; GSH, glutathione (reduced form); GSSG, glutathione disulfide (oxidized form); Put, putrescine; Cad, cadaverine; Spd, spermidine; Spm, spermine; IAA, indole-3-acetic acid; ABA, abscisic acid; SA, salicylic acid; JA, jasmonic acid; IPA, isopentenyl adenine; tZ, *trans*-zeatin; cZ; *cis*-zeatin; tZR, *trans*-zeatin riboside; cZR, *cis*-zeatin riboside; APRE, Albino Plant Regeneration Efficiency; GPRE, Green Plant Regeneration Efficiency; 1, 5, 8, analysed lines; T > C means that T and C form separate groups based on post-hoc test; A, B, and C reflect post-hoc groups.

**Table 5:** The arrangement of the Ridge regression analysis results illustrating how the analyzed biochemical variables and metAFLP quantitative characteristics impact APRE and GPRE.

				Ridge Regression Analysis					
				Common		General (Basic)		General (Extended)	
			Models	APRE ^1^	GPRE	APRE	GPRE	APRE	GPRE
			Statistics						
			Optimal lambda.	5.56	0.640	8.93	1.24	1.57	3.0
Classification			Intercept.	331.0	129.0	328.88	128.54	0.74	109.02
metAFLP characteristics			CHH_SV	−32.02	12.17	−25.97	10.13	−46.8	25.08
			CHH_DMV	−5.99	0	0	0	0	0
			CHH_DNMV	30.26	0.75	30.65	0	72.83	0
			CG_SV	30.97	−15.42	34.28	−14.14	86.39	−20.99
			CG_DMV	−145.39	25.34	−134.85	23.1	−105.8	0
			CG_DNMV	0	−18.4	0	−17.883	−38.33	−6.9
			CHG_SV	24.28	−7.87	21.31	−8.53	0	−6.81
			CHG_DMV	0	−34.71	0	−29.84	0	−15.95
			CHG_DNMV	41.034	14.51	21.64	8.74	0	0
Growth and Stress-Related Metabolites	Sulfur-containing metabolites ^2^		SAM ^2^	0	377.73	0	357.34	1922.24	331.27
			GSH	4.3	−2.93	0	−1.16	−7.53	0
			GSSG	0	0.183	0	0	0	0.02
	Polyamines ^3^		Put ^3^			0	0	0	0
			Cad			0	6.57	798.43	36.19
			Spd			0	1.0	0	0.74
			Spm			0.68	0	3.2	0.68
	Phytohormones ^4^		IAA ^4^					−0.16	−0.06
			ABA					0.056	0
			SA					0	0
			JA					0	0
			tZ					−0.56	0
			cZ					−0.23	0
			tZR					−25.33	0
			cZR					0	0
			IPA					0	0

^1^ APRE, Albino Plant Regeneration Efficiency; GPRE, Green Plant Regeneration Efficiency; ^2^ SAM, S-adenosyl-L-methionine; GSH, Glutathione (reduced form); GSSG, Glutathione Disulfide (oxidized form); ^3^ Put, putrescine; Cad, cadaverine; Spd, spermidine; Spm, spermine; ^4^ IAA, indole-3-acetic acid; ABA, abscisic acid; SA, salicylic acid; JA, jasmonic acid; tZ, *trans*-zeatin; cZ; *cis*-zeatin; tZR, *trans*-zeatin riboside; cZR, *cis*-zeatin riboside IPA, isopentenyl adenine.

## Data Availability

The original contributions presented in this study are included in the article. Further inquiries can be directed to the corresponding authors.

## Supplementary Materials

The following supporting information can be downloaded at: https://www.mdpi.com/article/10.3390/cells14151167/s1. Table S1: The parameters used to perform the Multiple Reaction Monitoring (MRM) quantification of phytohormones. Table S2: Data for metAFLP (SV, DMV, DNMV, D_Met and TCIV in CHH, CHG and CG sequence contexts) and regeneration parameters for all regenerants. Table S3: Data for growth and stress-related metabolites for all regenerants. Table S4: Descriptive statistics for metAFLP (SV, DMV, DNMV, D_Met, and TCIV in CHH, CHG, and CG sequence contexts) and biochemical characteristics.

## Abbreviations

The following abbreviations are used in this manuscript:

AAs	Amino acids
ABA	Abscisic acid
ADC-1	Arginine decarboxylase-1
AHC	Agglomerative hierarchical analysis
AIH	Agmatine iminohydrolase
APRE	Albino plant regeneration efficiency
Asn	Aspargine
Cad	Cadaverine
CG, CHG, and CHH	Methylation context
COX	Cytochrome *c* oxidase
CuAOx	Copper amine oxidase
cZ	cis-zeatin
cZR	cis-zeatin riboside
DMV	Demethylation
DNMV	De novo methylation
ETC	Electron transport chain
Gln	Glutamine
GPRE	Green plant regeneration efficiency
GSH	Glutathione (reduced form)
GSSG	Glutathione disulfide (oxidized form)
HPLC	High-pressure liquid chromatography
IAA	Indole-3-acetic acid
IM	Induction medium
IPA	Isopentenyladenine
JA	Jasmonic acid
Lys	Lysine
metAFLP	Methylation-sensitive amplified fragment length polymorphism
MRM	Multiple reaction monitoring
NAA	Naphtylo acetic acid
NEM	N-ethylmaleimide
ODC	Ornitine decarboxylase
PA	Polyamine
PCoA	Principal coordinated analysis
PCR	Polymerase chain reaction
PLP	Pyridoxal phosphate
PQH2	Plastoquinol
PSI, PSII	Photosystems I and II
Put	Putrescine
ROS	Reactive oxygen species
RP-HPLC	Reverse-phase high-pressure liquid chromatography
SA	Salicylic acid
SAM	S-adenosyl-L-methionine
Ser	Serine
SOD	Superoxide dismutase
Spd	Spermidine
SPE	Solid-phase extraction
Spm	Spermine
SV	Sequence variation
TCA	Trichloroacetic acid
TCIV	Tissue culture-induced variation
tZ	trans-zeatin
tZR	trans-zeatin riboside
UPGMA	Unweighted pair group method with arithmetic mean
