# Supplementary material for: Cu2+ and Zn2+ Ions Affecting Biochemical Paths and DNA Methylation of Rye (Secale cereale L.) Anther Culture Influencing Plant Regeneration Efficiency
Source: Cells. 2025 Jul 29;14(15):1167. doi: 10.3390/cells14151167 (PMC12345883; doi:10.3390/cells14151167)
Supplement: Supplementary file 1 [file cells-14-01167-s001.zip › cells-3763854-supplementary.pdf]

Table S1. The parameters used to perform the Multiple Reaction Monitoring (MRM) quantification of phytohormones.

Compound*	MRM 1	MRM 2
IAA	176.1 → 130.3	176.1 → 77.2
IPA	204.2 → 136.3	204.2 → 148.3
ABA	265.2 → 229.1	265.2 → 247.1
SA	139.0 → 121.0	139.0 → 39.0
JA	211.1 → 133.1	211.1 → 151.1
tZ	220.2 → 202.3	220.2 → 136.3
cZ	220.2 → 202.3	220.2 → 136.3
tZR	352.4 → 136.3	352.4 → 220.3
cZR	352.4 → 136.3	352.4 → 220.3

*Abbreviations used: IAA, indole-3-acetic acid; IPA, isopentenyl adenine; ABA, abscisic acid; SA, salicylic acid; JA, jasmonic acid; tZ, *trans*-zeatin; cZ, *cis*-zeatin; tZR, *trans*-zeatin riboside; cZR *cis*-zeatin riboside.

Table S2. Data for metAFLP (SV, DMV, DNMV, D_MET and TCIV in CHH, CHG and CG sequence contexts) and regeneration parameters for all regenerants.

Type of sample	Sample	metAFLP quantitative characteristics (%)*																				Regeneration	
		Sequence context															Total						
		CHH					CHG					CG											
		SV*	DMV	DNMV	TCIV	D_MET	SV	DMV	DNMV	TCIV	D_MET	SV	DMV	DNMV	TCIV	D_MET	SV	DMV	DNMV	TCIV	D_MET	APRE	GPRE
**Controls**	C1	2.586	3.978	1.945	8.158	-2.033	4.064	5.491	2.345	11.842	-3.146	2.677	4.04	2.611	9.737	-1.429	9.327	13.509	6.901	-6.608	29.737	10.00	3.33
	C1	3.117	4.205	3.472	10.345	-0.733	5.128	5.042	2.817	12.997	-2.226	2.416	4.091	3.134	10.08	-0.957	10.661	13.338	9.423	-3.916	33.422	10.00	3.75
**Treated**	T1	1.656	3.847	1.057	6.283	-2.79	2.78	5.71	2.616	10.995	-3.094	3.646	3.656	2.518	10.209	-1.138	8.082	13.214	6.191	-7.022	27.487	5.56	4.17
	T1	1.786	4.233	1.121	6.842	-3.111	2.807	5.501	2.491	10.789	-3.011	3.152	2.935	1.764	8.158	-1.171	7.744	12.669	5.376	-7.293	25.789	5.56	4.17
	T1	1.745	3.448	1.142	6.085	-2.305	2.382	5.683	2.292	10.317	-3.391	2.222	3.304	1.592	7.407	-1.711	6.349	12.434	5.026	-7.407	23.81	0.00	1.09
	T1	2.604	4.265	1.688	8.201	-2.577	4.2	5.125	2.558	11.905	-2.567	1.927	3.573	2.103	7.937	-1.47	8.73	12.963	6.349	-6.614	28.042	6.82	2.27
	T1	2.123	3.832	1.507	7.162	-2.325	3.562	4.996	3.171	11.671	-1.825	2.201	3.841	2.353	8.753	-1.488	7.886	12.669	7.031	-5.638	27.586	12.05	8.43
**Controls**	C5	3.562	1.409	1.822	6.631	0.413	3.208	2.329	1.11	6.631	-1.22	4.881	0.518	0.525	6.101	0.006	11.65	4.257	3.457	-0.8	19.363	433.33	13.33
	C5	3.136	1.771	1.342	6.101	-0.429	3.221	2.321	0.852	6.366	-1.469	4.881	0.518	0.526	6.101	0.008	11.237	4.61	2.72	-1.89	18.568	433.33	13.33
	C5	4.39	1.641	0.462	6.316	-1.18	3.173	2.306	1.39	6.842	-0.915	5.353	0.514	1.298	7.368	0.784	12.916	4.461	3.15	-1.312	20.526	433.33	13.33
	C5	3.841	1.648	1.004	6.349	-0.644	2.469	2.174	1.723	6.349	-0.451	5.162	0.776	0.78	6.878	0.004	11.472	4.598	3.506	-1.092	19.577	433.33	13.33
	C5	3.619	1.644	1.007	6.069	-0.637	4.22	2.202	2.378	8.707	0.176	5.638	0.627	1.619	8.179	0.993	13.477	4.473	5.005	0.532	22.955	433.33	13.33
	C5	2.919	1.556	1.746	6.085	0.189	2.92	2.088	1.39	6.349	-0.699	5.398	0.777	0.783	7.143	0.006	11.237	4.422	3.918	-0.504	19.577	433.33	13.33
	C5	3.135	2.032	1.086	6.085	-0.946	3.507	2.058	2.171	7.672	0.113	5.552	0.975	0.649	7.407	-0.326	12.195	5.064	3.905	-1.159	21.164	490.00	34.00
	C5	4.746	1.38	0.468	6.366	-0.912	4.227	2.102	1.961	8.223	-0.141	5.523	0.977	1.693	8.488	0.716	14.496	4.459	4.122	-0.337	23.077	490.00	34.00
	C5	3.154	1.493	1.884	6.349	0.392	4.124	2.12	1.514	7.672	-0.606	5.518	0.456	1.696	7.937	1.239	12.795	4.069	5.094	1.025	21.958	490.00	34.00
	C5	3.419	1.221	2.97	7.407	1.748	3.805	2.04	1.888	7.672	-0.152	5.349	0.518	2.336	8.466	1.818	12.572	3.779	7.193	3.414	23.545	490.00	34.00
	C5	3.439	1.226	2.71	7.181	1.484	3.874	1.932	1.713	7.447	-0.219	5.744	0.869	1.365	8.245	0.495	13.057	4.027	5.788	1.76	22.872	490.00	34.00
	C5	3.159	1.175	1.417	5.585	0.242	3.873	1.928	2.001	7.713	0.073	5.447	0.633	1.378	7.713	0.744	12.479	3.736	4.796	1.06	21.011	627.78	43.06
	C5	3.139	0.898	0.864	4.762	-0.034	3.769	1.816	1.394	6.878	-0.422	5.282	0.977	1.437	7.937	0.46	12.19	3.691	3.695	0.004	19.577	627.78	43.06
	C5	1.899	0.537	1.607	3.979	1.069	2.286	1.285	0.937	4.509	-0.348	2.333	0.262	0.525	3.183	0.264	6.518	2.084	3.069	0.985	11.671	627.78	43.06
	C5	2.164	0.804	0.538	3.43	-0.266	2.545	1.537	2.003	6.069	0.466	2.829	0.26	0.776	3.958	0.516	7.539	2.601	3.317	0.716	13.456	627.78	43.06
	C5	1.905	0.809	1.069	3.714	0.26	2.628	1.654	0.762	5.04	-0.892	2.325	0	1.579	3.979	1.579	6.859	2.463	3.41	0.947	12.732	627.78	43.06
	C5	2.044	0.632	0.609	3.209	-0.023	2.738	1.253	2.437	6.417	1.184	2.861	0	0.261	3.209	0.261	7.644	1.884	3.306	1.422	12.834	627.78	43.06
	C5	1.491	0.623	1.394	3.439	0.771	3.594	0.901	0.862	5.291	-0.04	3.574	0	0.525	4.233	0.525	8.659	1.524	2.78	1.256	12.963	627.78	43.06
	C5	1.909	0.268	1.332	3.457	1.064	2.566	0.758	0.15	3.457	-0.607	2.592	0	0.266	2.926	0.266	7.067	1.026	1.748	0.722	9.84	367.65	32.35
	C5	1.733	1.073	1.81	4.509	0.737	3.216	1.102	2.325	6.631	1.223	2.499	0.2	1.427	4.244	1.227	7.448	2.375	5.562	3.187	15.385	367.65	32.35
	C5	1.632	0.268	1.342	3.2	1.074	2.037	0.494	0.69	3.2	0.195	2.426	0.114	1.93	4.533	1.816	6.095	0.876	3.962	3.086	10.933	367.65	32.35
**Treated**	T5	1.217	0.632	1.144	2.941	0.513	2.501	1.256	1.367	5.08	0.111	3.391	0.264	0.527	4.278	0.263	7.109	2.152	3.039	0.887	12.299	326.47	22.06
	T5	2.046	0.362	0.608	2.949	0.245	2.586	1.378	1.985	5.898	0.607	4.181	0.264	0.263	4.826	-0.002	8.813	2.004	2.855	0.851	13.673	326.47	22.06
	T5	1.36	1.084	0.273	2.674	-0.811	1.76	1.364	2.254	5.348	0.89	3.679	0.264	0.261	4.278	-0.002	6.799	2.712	2.788	0.076	12.299	326.47	22.06

	T5	1.455	0.648	0.346	2.387	-0.303	3.867	0.642	1.412	5.836	0.77	3.318	0.264	1.046	4.775	0.783	8.64	1.554	2.804	1.25	12.997	326.47	22.06
	T5	2.178	0.535	1.336	3.968	0.801	2.978	1.107	0.996	5.026	-0.111	4.183	0.374	0.599	5.291	0.225	9.339	2.016	2.931	0.915	14.286	566.04	49.06
	T5	2.392	0.446	1.723	4.497	1.277	1.728	1.191	1.061	3.968	-0.13	3.38	0	1.57	5.026	1.57	7.5	1.638	4.355	2.717	13.492	566.04	49.06
	T5	2.716	0.268	1.072	3.979	0.803	2.436	1.109	1.017	4.509	-0.092	4.401	0	2.101	6.631	2.101	9.553	1.377	4.189	2.813	15.119	566.04	49.06
	T5	2.545	0.089	0.868	3.457	0.778	1.357	0.848	1.271	3.457	0.423	4.199	0	0.525	4.787	0.525	8.101	0.937	2.664	1.727	11.702	566.04	49.06
	T5	2.57	0.361	0.881	3.743	0.52	1.787	1.345	2.253	5.348	0.908	3.94	0	1.568	5.615	1.568	8.297	1.706	4.702	2.996	14.706	566.04	49.06
	T5	2.754	0.269	2.444	5.305	2.175	3.927	1.465	2.372	7.692	0.907	4.846	0.261	1.553	6.897	1.292	11.528	1.996	6.37	4.375	19.894	566.04	49.06
	T5	1.644	0.269	1.071	2.91	0.802	3.685	1.55	1.216	6.349	-0.334	4.081	0.26	1.568	6.085	1.308	9.41	2.079	3.855	1.776	15.344	566.04	49.06
	T5	2.466	0.534	2.964	5.82	2.43	3.617	0.93	1.841	6.349	0.912	3.827	0.262	2.077	6.349	1.815	9.91	1.726	6.883	5.157	18.519	566.04	49.06
	T5	2.442	0.533	1.606	4.485	1.073	2.871	0.991	1.451	5.277	0.459	3.663	0.373	1.374	5.541	1.001	8.975	1.897	4.431	2.534	15.303	566.04	49.06
	T5	3.655	1.167	0.33	5.053	-0.836	2.223	1.214	1.65	5.053	0.436	4.161	0	2.355	6.649	2.355	10.039	2.381	4.336	1.955	16.755	566.04	49.06
	T5	2.883	0.628	1.135	4.521	0.507	3.853	1.226	1.091	6.117	-0.135	3.911	0.375	1.654	6.117	1.278	10.647	2.229	3.88	1.651	16.755	566.04	49.06
	T5	3.969	0.896	0.595	5.305	-0.301	3.637	1.282	0.963	5.836	-0.319	4.676	0.374	2.177	7.427	1.803	12.282	2.551	3.735	1.183	18.568	566.04	49.06
	T5	3.573	0.537	1.615	5.585	1.078	3.346	1.024	1.21	5.585	0.186	3.415	0.376	1.126	5.053	0.75	10.334	1.938	3.951	2.014	16.223	566.04	49.06
	T5	2.589	0.893	0.328	3.714	-0.565	3.386	1.018	1.219	5.57	0.201	3.919	0.375	0.861	5.305	0.486	9.894	2.286	2.408	0.122	14.589	566.04	49.06
	T5	2.869	0.626	3.032	6.366	2.406	3.597	1.197	1.832	6.631	0.635	3.398	0.374	1.376	5.305	1.002	9.865	2.198	6.24	4.042	18.302	566.04	49.06
	T5	3.783	0.986	0.932	5.556	-0.053	2.952	1.603	2.321	6.878	0.718	3.411	0.372	1.366	5.291	0.994	10.145	2.96	4.619	1.659	17.725	566.04	49.06
	T5	3.66	0.804	2.603	6.878	1.799	3.779	1.342	1.774	6.878	0.432	4.147	0.373	2.153	6.878	1.78	11.586	2.52	6.53	4.01	20.635	566.04	49.06
	T5	3.681	0.624	1.402	5.556	0.779	3.493	1.081	1.516	6.085	0.435	3.648	0.374	1.377	5.556	1.003	10.821	2.079	4.296	2.217	17.196	566.04	49.06
	T5	3.574	0.597	2.121	6.183	1.524	2.171	1.438	1.234	4.839	-0.204	3.945	0	2.124	6.183	2.124	9.69	2.035	5.479	3.443	17.204	704.29	18.57
	T5	3.185	1.04	1.235	5.333	0.195	3.216	1.106	1.28	5.6	0.174	3.356	0	1.316	4.8	1.316	9.757	2.146	3.831	1.685	15.733	704.29	18.57
	T5	3.693	0.653	1.167	5.333	0.514	4.293	0.871	2.094	7.2	1.223	4.852	0	1.044	6.133	1.044	12.838	1.524	4.305	2.781	18.667	704.29	18.57
	T5	4.361	0.741	0.676	5.6	-0.065	3.944	0.605	1.033	5.6	0.428	3.585	0	1.056	4.8	1.056	11.89	1.346	2.764	1.418	16	704.29	18.57
	T5	3.793	0.706	0.914	5.249	0.208	4.097	0.871	1.121	6.037	0.25	4.533	0.26	2.076	7.087	1.816	12.423	1.837	4.112	2.275	18.373	704.29	18.57
	T5	2.644	0.478	3.448	6.417	2.97	1.631	1.432	3.111	6.15	1.679	3.683	0	3.627	7.487	3.627	7.958	1.91	10.186	8.276	20.053	704.29	18.57
	T5	3.477	0.475	2.037	5.851	1.562	2.734	1.399	2.004	6.117	0.604	4.14	0	2.085	6.383	2.085	10.351	1.874	6.125	4.251	18.351	704.29	18.57
	T5	3.645	0.919	2.745	7.162	1.826	3.466	1.017	0.584	5.04	-0.433	4.611	0.263	1.055	6.101	0.792	11.722	2.198	4.383	2.185	18.302	704.29	18.57
	T5	4.009	0.686	3.577	8.043	2.891	3.425	1.32	2.496	7.239	1.175	3.958	0.113	3.473	7.775	3.36	11.392	2.119	9.545	7.426	23.056	704.29	18.57
	T5	3.505	0.474	2.556	6.383	2.082	3.568	1.226	1.072	5.851	-0.154	4.105	0.263	1.315	5.851	1.053	11.179	1.963	4.943	2.98	18.085	704.29	18.57
	T5	2.997	0.757	3.776	7.297	3.019	3.381	1.349	3.428	8.108	2.079	4.18	0.533	3.654	8.649	3.121	10.558	2.638	10.858	8.22	24.054	704.29	18.57
	T5	3.778	0.745	0.403	4.787	-0.342	3.483	1.37	0.478	5.319	-0.893	4.108	0	0.529	4.787	0.529	11.369	2.115	1.41	-0.705	14.894	704.29	18.57
	T5	2.982	0.476	0.946	4.278	0.47	3.998	1.052	0.877	5.882	-0.175	4.109	0	0.265	4.545	0.265	11.09	1.528	2.088	0.56	14.706	427.14	12.86
	T5	2.897	0.361	1.135	4.267	0.774	4.07	1.586	0.795	6.4	-0.791	3.839	0.262	1.587	5.867	1.325	10.806	2.21	3.517	1.308	16.533	427.14	12.86
	T5	3.159	0.36	1.67	5.053	1.311	3.82	1.401	0.927	6.117	-0.474	4.095	0	0.527	4.787	0.527	11.073	1.76	3.124	1.364	15.957	427.14	12.86
	T5	2.879	0.898	0.864	4.521	-0.034	3.681	1.305	0.878	5.851	-0.428	3.331	0	0.791	4.255	0.791	9.891	2.204	2.533	0.329	14.628	427.14	12.86
	T5	2.609	0.36	1.134	3.989	0.774	3.897	1.518	1.018	6.383	-0.5	3.58	0.262	1.58	5.585	1.318	10.085	2.14	3.732	1.592	15.957	427.14	12.86
	C8	3.525	0.718	2.681	6.824	1.963	2.163	0.786	0.687	3.675	-0.099	2.311	0.521	0.782	3.675	0.261	7.999	2.025	4.149	2.125	14.173	392.50	157.50
**Control**	C8	3.598	0.997	1.115	5.585	0.119	2.896	0.896	1.214	5.053	0.318	2.139	0.64	0.6	3.457	-0.04	8.633	2.533	2.93	0.397	14.096	392.50	157.50
	C8	3.579	1.265	0.308	5.053	-0.957	2.374	0.895	1.487	4.787	0.593	2.266	0.728	0.395	3.457	-0.333	8.219	2.888	2.191	-0.697	13.298	392.50	157.50
	C8	3.575	0.996	0.309	4.787	-0.687	2.254	0.808	1.693	4.787	0.886	2.153	0.641	0.336	3.191	-0.305	7.983	2.444	2.339	-0.106	12.766	392.50	157.50

	C8	3.303	1.264	0.309	4.787	-0.955	2.267	0.806	1.693	4.787	0.888	2.413	0.641	0.336	3.457	-0.305	7.983	2.71	2.339	-0.371	13.032	392.50	157.50
	C8	3.751	0.963	0.736	5.348	-0.227	2.275	0.81	1.429	4.545	0.619	2.687	0.379	0.339	3.476	-0.04	8.713	2.152	2.504	0.352	13.369	392.50	157.50
	C8	3.755	1.503	0.735	5.882	-0.769	2.393	0.894	1.222	4.545	0.328	2.684	0.379	0.34	3.476	-0.039	8.832	2.776	2.296	-0.48	13.904	392.50	157.50
	C8	4	0.956	0.461	5.319	-0.496	2.183	0.694	1.084	3.989	0.389	2.716	0.616	0.322	3.723	-0.295	8.899	2.267	1.866	-0.401	13.032	242.50	162.50
	C8	3.785	0.978	0.303	4.987	-0.675	1.942	0.349	1.109	3.412	0.76	3.059	0.697	0.375	4.199	-0.323	8.786	2.025	1.787	-0.237	12.598	242.50	162.50
	C8	3.89	0.568	1.708	6.053	1.14	2.43	0.79	0.952	4.211	0.162	2.681	0.609	0.319	3.684	-0.291	9.002	1.967	2.978	1.011	13.947	242.50	162.50
	C8	3.535	0.715	0.569	4.724	-0.146	2.461	0.349	1.106	3.937	0.757	2.673	0.349	0.317	3.412	-0.031	8.67	1.412	1.991	0.579	12.073	242.50	162.50
	C8	3.962	1.336	0.628	5.789	-0.707	3.177	0.547	0.979	4.737	0.431	2.915	0.611	0.318	3.947	-0.292	10.054	2.494	1.926	-0.568	14.474	242.50	162.50
	C8	3.782	0.931	0.986	5.585	0.056	2.49	0.669	1.592	4.787	0.923	2.597	0.579	0.469	3.723	-0.109	8.869	2.178	3.048	0.87	14.096	242.50	162.50
	C8	3.645	1.06	0.804	5.376	-0.256	3.008	1.022	1.862	5.914	0.84	2.35	0.849	1.529	4.839	0.68	9.003	2.931	4.194	1.263	16.129	242.50	162.50
	C8	3.546	1.209	0.447	5.08	-0.762	3.001	1.011	1.306	5.348	0.295	2.523	0.466	0.662	3.743	0.196	9.07	2.687	2.415	-0.272	14.171	242.50	162.50
	C8	3.546	0.939	0.722	5.08	-0.217	2.993	1.017	1.832	5.882	0.815	2.264	0.731	0.396	3.476	-0.335	8.802	2.687	2.95	0.263	14.439	242.50	162.50
	C8	3.645	1.332	0.804	5.645	-0.528	3.008	1.017	1.842	5.914	0.825	2.35	0.583	0.473	3.495	-0.11	9.003	2.931	3.119	0.188	15.054	242.50	162.50
	C8	3.414	1.116	0.931	5.333	-0.185	2.994	1.008	1.827	5.867	0.819	2.443	0.352	0.582	3.467	0.23	8.851	2.476	3.34	0.863	14.667	242.50	162.50
	C8	3.414	1.116	0.659	5.067	-0.456	2.994	1.011	1.837	5.867	0.826	2.443	0.616	0.844	4	0.228	8.851	2.743	3.34	0.597	14.933	242.50	162.50
	C8	3.427	1.12	1.203	5.6	0.083	3.376	1.361	1.876	6.667	0.515	2.433	0.351	0.32	3.2	-0.031	9.236	2.832	3.399	0.567	15.467	975.00	100.00
	C8	3.602	1.045	0.791	5.319	-0.254	2.895	0.581	1.27	4.787	0.689	2.254	0.73	0.396	3.457	-0.334	8.751	2.356	2.457	0.101	13.564	975.00	100.00
	C8	3.621	1.057	0.528	5.094	-0.528	2.612	0.928	1.78	5.362	0.852	2.163	0.645	0.339	3.217	-0.306	8.396	2.63	2.647	0.017	13.673	975.00	100.00
	T8	3.515	1.204	2.054	6.649	0.85	2.633	1.154	0.95	4.787	-0.204	2.071	0.263	1.581	3.989	1.318	8.219	2.622	4.585	1.963	15.426	207.41	72.22
	T8	3.479	0.925	2.039	6.332	1.114	2.396	0.814	0.977	4.222	0.164	2.317	0.523	1.306	4.222	0.783	8.192	2.262	4.322	2.061	14.776	207.41	72.22
	T8	3.493	0.921	0.702	5.013	-0.219	2.81	0.462	0.397	3.694	-0.065	2.564	0.263	0.526	3.43	0.263	8.866	1.646	1.625	-0.021	12.137	207.41	72.22
	T8	3.214	0.924	0.441	4.497	-0.483	2.294	0.464	0.934	3.704	0.471	2.4	0.376	1.123	3.968	0.747	7.907	1.764	2.499	0.735	12.169	207.41	72.22
	T8	3.634	0.57	0.379	4.485	-0.19	2.537	0.727	0.663	3.958	-0.064	2.314	0.262	0.524	3.166	0.262	8.485	1.558	1.566	0.008	11.609	207.41	72.22
	T8	3.497	0.659	0.975	5.013	0.317	2.922	0.814	1.25	5.013	0.436	2.418	0.349	1.1	3.958	0.751	8.837	1.822	3.325	1.504	13.984	207.41	72.22
	T8	3.401	0.543	1.426	5.263	0.882	2.652	0.814	0.978	4.474	0.164	2.305	0.522	1.042	3.947	0.52	8.358	1.88	3.446	1.566	13.684	207.41	72.22
	T8	3.142	0.812	1.164	5.013	0.352	2.756	0.925	1.061	4.749	0.137	2.822	0.261	1.305	4.485	1.044	8.72	1.998	3.531	1.533	14.248	207.41	72.22
	T8	3.041	0.729	0.575	4.255	-0.153	2.625	0.92	0.978	4.521	0.058	2.849	0.263	1.317	4.521	1.053	8.515	1.912	2.871	0.958	13.298	207.41	72.22
	T8	3.045	0.462	0.574	3.989	0.113	2.743	1.277	0.77	4.787	-0.507	2.845	0.263	1.32	4.521	1.057	8.633	2.001	2.664	0.663	13.298	207.41	72.22
**Treated**	T8	3.405	0.579	2.001	5.867	1.423	2.741	1.282	1.026	5.067	-0.256	2.853	0.526	1.32	4.8	0.793	8.999	2.387	4.347	1.96	15.733	207.41	72.22
	T8	3.314	1	0.306	4.521	-0.694	2.618	1.185	0.708	4.521	-0.477	2.849	0.526	0.792	4.255	0.267	8.781	2.71	1.807	-0.903	13.298	207.41	72.22
	T8	3.312	0.998	0.307	4.521	-0.692	2.537	1.07	0.898	4.521	-0.172	2.666	0.376	0.602	3.723	0.227	8.515	2.444	1.807	-0.637	12.766	207.41	72.22
	T8	3.316	0.73	1.377	5.319	0.647	2.655	1.161	0.687	4.521	-0.475	2.663	0.376	1.132	4.255	0.756	8.633	2.267	3.196	0.929	14.096	207.41	72.22
	T8	3.39	0.845	0.382	4.521	-0.463	2.54	1.07	0.632	4.255	-0.439	2.851	0.263	0.793	3.989	0.53	8.781	2.178	1.807	-0.371	12.766	207.41	72.22
	T8	3.509	0.972	0.735	5.108	-0.237	2.646	1.198	0.714	4.57	-0.484	3.028	0.493	0.953	4.57	0.46	9.182	2.663	2.402	-0.26	14.247	207.41	72.22
	T8	1.703	0.964	0.819	3.439	-0.145	2.138	0.716	0.566	3.439	-0.15	0.892	0.613	0.585	2.116	-0.028	4.733	2.293	1.969	-0.323	8.995	207.41	72.22
	T8	2.011	0.672	1.068	3.704	0.396	1.864	0.716	0.563	3.175	-0.153	0.593	0.375	0.338	1.323	-0.037	4.468	1.764	1.969	0.206	8.201	207.41	72.22
	T8	1.971	0.7	1.089	3.714	0.389	1.729	0.808	0.623	3.183	-0.185	0.856	0.639	0.339	1.857	-0.3	4.556	2.147	2.05	-0.097	8.753	207.41	72.22
	T8	1.632	0.906	1.228	3.723	0.321	1.734	0.808	0.623	3.191	-0.185	0.597	0.641	0.34	1.596	-0.301	3.964	2.356	2.191	-0.165	8.511	207.41	72.22
	T8	1.039	0.92	0.964	2.902	0.043	1.689	0.351	0.058	2.111	-0.293	0.333	0.639	0.075	1.055	-0.563	3.062	1.91	1.097	-0.812	6.069	378.33	151.67
	T8	1.491	0.541	1.416	3.421	0.876	1.417	0.351	0.317	2.105	-0.034	0.333	0.637	0.075	1.053	-0.562	3.241	1.529	1.809	0.28	6.579	378.33	151.67

T8	1.751	0.54	2.198	4.462	1.658	1.264	0.437	0.114	1.837	-0.323	0.259	0.523	0.262	1.05	-0.26	3.275	1.5	2.575	1.075	7.349	378.33	151.67
T8	0.796	0.573	0.729	2.089	0.156	1.039	0.261	0	1.305	-0.261	0.258	0.521	0	0.783	-0.521	2.093	1.355	0.729	-0.626	4.178	378.33	151.67
T8	1.756	0.54	1.677	3.937	1.137	1.527	0.437	0.375	2.362	-0.063	0.517	0.523	0.523	1.575	0	3.8	1.5	2.575	1.075	7.874	378.33	151.67
T8	1.553	0.6	0.748	2.88	0.148	1.296	0.261	0	1.571	-0.261	0.517	0.261	0	0.785	-0.261	3.366	1.122	0.748	-0.374	5.236	378.33	151.67
T8	1.555	0.338	0.748	2.618	0.411	1.407	0.35	0.058	1.832	-0.292	0.258	0.522	0.262	1.047	-0.26	3.22	1.209	1.068	-0.141	5.497	378.33	151.67
T8	1.232	0.494	0.682	2.375	0.189	2.134	0.715	0.565	3.43	-0.15	0.516	0.262	0.525	1.319	0.263	3.882	1.47	1.772	0.302	7.124	378.33	151.67
T8	1.23	0.762	0.685	2.646	-0.077	1.728	0.803	0.624	3.175	-0.18	0.334	0.375	0.338	1.058	-0.037	3.292	1.94	1.646	-0.294	6.878	378.33	151.67
T8	1.494	0.495	0.684	2.646	0.189	1.345	0.719	0.565	2.646	-0.154	0.336	0.638	0.338	1.323	-0.3	3.175	1.852	1.587	-0.265	6.614	378.33	151.67
T8	1.844	0.611	1.029	3.448	0.418	1.344	0.72	0.565	2.653	-0.156	0.336	0.64	0.339	1.326	-0.3	3.524	1.97	1.933	-0.038	7.427	378.33	151.67
T8	1.227	0.228	0.685	2.116	0.457	1.469	0.544	0.624	2.646	0.08	0.596	0.639	0.338	1.587	-0.301	3.292	1.411	1.646	0.235	6.349	378.33	151.67
T8	1.043	0.612	0.497	2.128	-0.116	1.628	0.721	0.569	2.926	-0.152	0.673	0.49	0.416	1.596	-0.075	3.343	1.824	1.482	-0.342	6.649	378.33	151.67
T8	1.318	0.876	0.761	2.91	-0.116	2.51	0.804	0.623	3.968	-0.181	0.258	0.525	0.263	1.058	-0.262	4.086	2.205	1.646	-0.558	7.937	378.33	151.67
T8	1.85	0.876	0.494	3.175	-0.382	1.976	0.804	0.624	3.439	-0.179	0.259	0.525	0.263	1.058	-0.262	4.086	2.205	1.382	-0.823	7.672	378.33	151.67
T8	2.156	0.875	0.76	3.704	-0.115	3.723	0.716	0.564	5.026	-0.152	1.528	0.525	0.263	2.381	-0.262	7.407	2.116	1.587	-0.529	11.111	378.33	151.67
T8	1.855	0.344	2.383	4.509	2.039	1.983	0.811	1.402	4.244	0.591	0.334	0.639	0.859	1.857	0.22	4.172	1.794	4.644	2.85	10.61	378.33	151.67
T8	2.062	0.496	0.686	3.183	0.19	2.648	0.72	0.568	3.979	-0.152	0.406	0.489	0.678	1.592	0.189	5.116	1.705	1.933	0.227	8.753	378.33	151.67
T8	1.24	0.495	0.418	2.116	-0.077	2.514	0.805	0.627	3.968	-0.178	0.332	0.375	0.601	1.323	0.226	4.086	1.675	1.646	-0.029	7.407	378.33	151.67
T8	0.78	0.612	0.498	1.862	-0.114	2.688	0.723	0.835	4.255	0.112	0.407	0.754	0.414	1.596	-0.34	3.875	2.09	1.748	-0.342	7.713	378.33	151.67
T8	1.126	0.729	0.575	2.4	-0.154	1.892	0.722	0.571	3.2	-0.152	0.411	0.492	0.417	1.333	-0.075	3.429	1.943	1.562	-0.381	6.933	378.33	151.67
T8	1.853	0.609	0.495	2.918	-0.114	1.867	0.454	0.568	2.918	0.114	0.335	0.377	0.339	1.061	-0.038	4.055	1.44	1.402	-0.038	6.897	378.33	151.67
T8	1.853	1.142	0.495	3.448	-0.647	1.867	0.451	0.568	2.918	0.116	0.335	0.377	0.339	1.061	-0.038	4.055	1.97	1.402	-0.568	7.427	378.33	151.67
T8	1.316	0.878	0.495	2.653	-0.382	2.145	0.716	0.568	3.448	-0.149	0.593	0.376	0.339	1.326	-0.037	4.055	1.97	1.402	-0.568	7.427	378.33	151.67
T8	1.502	0.759	0.42	2.646	-0.339	1.868	0.452	1.098	3.439	0.646	0.334	0.376	0.598	1.323	0.222	3.704	1.587	2.116	0.529	7.407	378.33	151.67
T8	1.506	0.494	0.418	2.381	-0.075	2.13	0.452	0.566	3.175	0.115	0.333	0.113	0.338	0.794	0.225	3.968	1.058	1.323	0.265	6.349	378.33	151.67
T8	1.04	0.876	0.495	2.387	-0.38	1.618	0.454	0.568	2.653	0.114	0.335	0.641	0.339	1.326	-0.302	2.994	1.97	1.402	-0.568	6.366	378.33	151.67
T8	1.583	0.609	0.495	2.653	-0.114	1.872	0.452	0.568	2.918	0.115	0.335	0.113	0.339	0.796	0.226	3.789	1.175	1.402	0.227	6.366	378.33	151.67

* SV, sequence variation; DMV, demethylation: DNMV, *de novo* methylation; D_MET, change in methylation; CHH, CHG, CG sequence contexts; C1, C5, C8, lines of regenerants obtained on a control medium; T1, T5, T8, lines of regenerants obtained on a tested media T1 and T5 with Cu^{2+} ions, T8 with Zn^{2+} ions; Regeneration efficiency, number of regenerants obtained per 100 plated anthers; APRE, albino plant regeneration efficiency; GPRE, green plant regeneration efficiency;

Table S3. Data for growth and stress-related metabolites for all regenerants.

Type of sample	Sample	Growth and Stress-Related Metabolites															
		**Sulfur-containing metabolites** (μmol g^{-1})			**Polyamines** (μg g^{-1})				**Phytohormones** (ng g^{-1})								
		**SAM**	**GSH**	**GSSG**	**Put**	**Cad**	**Spd**	**Spm**	**IAA**	**IPA**	**ABA**	**SA**	**JA**	**tZ**	**cZ**	**tZR**	**cZR**
**Controls**	C1	0.06	5.83	29.03	4.09	0.19	7.37	12.85	27.93	1368.52	1051.14	1240.42	10.67	27.24	1.02	23.18	0.63
	C1	0.04	1.89	11.00													
	T1	0.02	0.58	5.66	1.02	1.03	1.76	0.94									
	T1																
**Treated**	T1	0.01	1.75	31.15	3.63	0.25	2.67	2.18	31.53	160.40	1636.59	566.45	8.60	44.12	0.36	20.57	0.78
	T1	0.05	1.39	22.69													
	T1	0.10	2.73	41.13	2.03	0.29	4.02	2.81									
	C5	0.02	2.11	52.73	0.79	0.16	1.23	4.34	172.12	32.29	659.21	536.13	8.55	10.04	0.40	73.38	1.02
	C5	0.02	1.09	19.23	0.46	0.14	1.36	5.54	175.03	159.01	2165.39	373.39	7.48	7.06	0.34	19.26	0.63
	C5	0.08	7.91	91.79	1.96	0.13	1.50	5.01									
	C5	0.03	2.08	34.24	0.57	0.11	1.51	4.93									
	C5	0.02	0.73	11.62	2.46	0.08	2.85	6.11	64.05	1051.34	2081.32	925.70	17.61	13.80	0.40	31.40	0.59
	C5	0.03	2.53	40.40	1.39	0.13	1.92	4.18									
	C5	0.02	2.14	28.33	2.67	0.24	2.90	4.20									
	C5				3.12	0.11	4.04	6.91	18.91	542.61	284.07	309.64	5.99	3.54	0.49	7.46	0.11
	C5	0.01	1.71	26.97													
	C5	0.03	1.17	11.37	0.76	0.09	1.01	3.08									
**Controls**	C5	0.05	2.60	32.50	1.06	0.03	1.50	7.09	29.02	544.31	708.40	216.15	34.53	32.63	1.19	348.75	0.53
	C5	0.02	1.41	23.38	2.37	0.24	3.84	9.66	24.62	2010.26	2403.13	815.23	15.67	10.49	1.19	14.53	0.50
	C5	0.07	5.21	45.20	2.19	0.10	2.98	5.64									
	C5				1.77	0.21	1.56	8.09									
	C5	0.03	2.33	29.28	2.13	0.21	3.69	7.79									
	C5	0.02	1.15	19.87	0.41	0.14	1.08	5.51									
	C5																
	C5	0.03	2.66	24.71	4.03	0.18	4.24	4.81	18.01	2481.87	876.49	1531.78	16.09	9.85	0.77	17.67	0.27
	C5	0.04	1.14	17.24													
	C5				14.98	0.19	37.36	41.28	39.21	1034.67	1364.36	1538.67	13.58	14.97	0.88	20.89	0.35
	C5	0.03	3.41	24.33	2.93	0.26	5.68	8.13									
	T5				0.18	0.20	0.53	3.47									
	T5				0.25	0.04	0.57	8.90									
**Treated**	T5	0.02	0.96	18.33	0.76	0.04	1.77	3.78									
	T5	0.02	0.99	5.10	1.41	0.14	1.38	6.45	24.68	758.16	1071.79	625.95	7.79	5.56	0.69	12.64	0.26
	T5	0.03	1.48	27.21	1.61	0.30	3.09	4.76	84.90	118.58	1183.43	725.29	44.72	18.56	0.25	22.81	0.47

	T5	0.02	0.89	13.14	0.17	0.16	0.75	8.09									
	T5	0.03	2.21	45.77	1.09	0.20	2.30	13.30	56.39	1513.76	2398.78	831.17	9.95	5.64	0.21	9.32	0.34
	T5	0.02	2.09	41.51	0.22	0.37	1.17	9.62									
	T5	0.01	1.10	15.26	1.77	0.17	2.32	8.09									
	T5	0.03	2.34	24.25	2.80	0.26	4.84	7.57									
	T5				1.63	0.10	3.69	3.84									
	T5	0.03	0.63	7.77	2.91	0.19	3.47	5.48	52.89	742.16	3527.77	968.56	29.49	33.35	0.54	184.38	0.62
	T5																
	T5	0.02	1.33	19.70	34.33	0.44	6.24	4.34									
	T5	0.04	1.80	20.04	0.34	0.09	0.64	5.02									
	T5	0.04	2.04	17.53	0.56	0.09	0.55	4.04									
	T5	0.02	1.16	18.29	0.66	0.10	1.33	4.65									
	T5	0.03	1.51	16.97	0.42	0.04	0.68	5.62									
	T5	0.01	0.68	5.05	0.51	0.08	1.39	6.56									
	T5	0.02	1.84	28.50	2.52	0.22	1.38	1.11									
	T5	0.04	2.44	8.95					39.94	809.20	1672.28	822.66	20.85	11.53	2.82	30.75	0.36
	T5	0.03	1.33	14.66													
	T5																
	T5	0.04	2.35	11.88	6.97	0.10	5.40	4.24									
	T5	0.02	1.59	18.74	3.99	0.23	5.16	3.34									
	T5																
	T5	0.05	0.82	13.59	6.48	0.27	3.57	3.79									
	T5	0.02	1.22	24.98	2.79	0.09	3.73	8.96									
	T5	0.02	1.05	19.70	1.02	0.06	2.18	9.41									
	T5	0.01	0.79	5.55	1.42	0.10	1.92	7.56									
	T5																
	T5				0.54	0.09	0.41	8.14									
	T5				1.80	0.13	3.05	3.49	29.96	147.36	1299.18	362.52	31.78	69.13	0.90	290.10	2.94
	T5	0.04	0.84	12.51	1.70	0.27	2.13	3.08									
	T5				0.54	0.05	0.91	10.95	50.41	448.21	1820.13	804.01	16.97	17.01	0.34	66.97	0.33
	T5	0.02	2.02	21.60	1.91	0.24	3.11	3.21									
	T5	0.03	1.85	23.29	1.55	0.11	1.40	4.82									
	T5	0.01	1.06	19.67	1.88	0.19	3.34	3.54									
	T5	0.03	2.12	14.71	3.16	0.19	4.80	9.15									
**Control**	C8	0.04	2.09	44.57													
	C8	0.05	0.87	18.59	13.95	0.16	45.06	41.69	60.17	787.40	1001.07	1692.27	9.41	4.05	0.75	5.09	0.14
	C8				8.77	0.15	28.38	25.78	103.72	775.81	1710.42	1188.40	22.32	23.13	0.75	32.10	0.62
	C8	0.07	1.70	19.21	0.98	0.23	2.89	3.17									
	C8	0.07	1.80	27.63	1.39	0.46	3.59	2.29	86.37	542.39	657.30	1223.30	38.23	26.38	3.14	47.78	0.62
	C8				1.76	0.16	2.43	2.33									

	C8	0.09	2.97	33.61	1.54	0.23	3.08	2.82	121.10	540.52	898.35	1209.62	24.68	22.37	2.41	63.84	0.33
	C8	0.02	1.24	24.28	1.35	0.20	1.50	1.91									
	C8				2.45	0.09	2.76	2.57	37.86	1022.32	1135.16	1297.98	28.00	15.26	0.95	39.36	0.25
	C8	0.04	2.75	50.58	1.87	0.12	3.26	3.31	86.32	1405.60	2000.25	695.05	23.45	11.28	1.35	29.19	0.39
	C8	0.04	1.76	28.04	0.92	0.10	2.20	4.12									
	C8	0.02	0.88	13.08	0.71	0.09	0.82	2.52									
	C8	0.05	1.27	22.91	13.13	0.22	34.22	39.67	27.37	3430.25	2491.62	1637.76	36.12	41.81	13.61	168.03	8.62
	C8	0.04	1.01	20.21	10.63	0.22	27.89	24.94	176.87	460.31	2179.77	1158.32	13.37	13.33	0.93	17.70	0.36
	C8	0.03	0.68	10.24	14.96	0.17	43.75	31.94	168.89	301.42	1599.08	1224.20	9.88	15.32	0.52	14.77	0.34
	C8	0.06	1.36	26.74	1.50	0.17	3.39	3.64	45.77	599.31	1149.94	1191.17	6.24	16.02	0.26	8.44	0.29
	C8	0.05	1.19	15.10	1.08	0.14	2.78	2.60									
	C8	0.08	1.51	17.28	2.34	0.36	5.80	5.66									
	C8																
	C8	0.06	1.43	26.01	9.52	0.11	26.38	29.67									
	C8	0.05	1.11	20.81	0.83	0.20	2.44	1.78									
	C8				1.86	0.28	3.17	2.52									
	T8	0.03	1.69	45.55	3.09	0.09	4.07	3.53									
	T8	0.02	0.98	26.30	2.26	0.09	2.77	3.45									
	T8	0.00	1.48	24.78													
	T8	0.04	2.34	35.17	2.43	0.07	2.61	7.59									
	T8	0.02	0.80	14.05	2.44	0.16	2.44	5.58									
	T8	0.04	1.75	22.71	2.97	0.04	4.14	4.89									
	T8	0.03	1.34	28.25	1.64	0.22	1.58	1.58									
	T8	0.05	0.88	13.73	2.80	0.17	3.56	2.73	79.82	313.83	2413.18	1149.82	53.91	65.03	1.09	199.05	0.60
	T8	0.03	0.55	6.73	2.36	0.17	3.30	3.37									
	T8	0.06	1.44	30.60	1.96	0.28	3.58	3.92									
	T8				3.18	0.09	3.00	3.11									
	T8				0.86	0.12	4.00	2.47									
**Treated**	T8	0.05	1.41	20.43	2.38	0.12	4.10	2.74	413.27	2965.87	2156.36	2996.88	32.71	5.56	3.83	13.94	0.21
	T8				2.07	0.23	3.31	1.56	115.94	688.93	1555.96	737.07	43.19	21.21	5.44	114.21	0.23
	T8	0.07	1.77	21.04	3.36	0.22	4.15	2.72									
	T8	0.05	1.28	17.93	1.61	0.14	3.05	1.92									
	T8	0.03	0.91	4.97	3.10	0.34	4.67	2.12									
	T8	0.06	1.38	15.68	1.36	0.29	3.14	1.33									
	T8				1.65	0.20	3.16	2.99	58.76	600.01	1631.31	471.48	60.15	17.28	8.07	36.36	0.34
	T8																
	T8	0.02	1.11	21.04	1.06	0.10	1.51	1.83									
	T8	0.02	0.76	16.46	3.75	0.07	5.04	4.85									
	T8	0.03	1.45	21.26	1.47	0.16	2.29	2.68									
	T8	0.03	1.57	24.43	1.19	0.15	2.46	1.97									

	T8	0.06	2.26	41.93	2.45	0.10	3.64	4.13									
	T8				2.07	0.04	4.43	4.96									
	T8				2.37	0.12	3.34	3.44									
	T8	0.06	1.72	25.61	2.33	0.10	2.45	2.76									
	T8	0.02	0.65	8.61	1.68	0.14	2.32	2.46									
	T8				0.93	0.07	3.71	4.41									
	T8				1.60	0.17	3.13	2.41	86.33	807.24	1386.04	606.80	2.80	2.52	1.95	5.15	0.24
	T8																
	T8				2.65	0.21	3.06	1.97									
	T8				1.38	0.14	4.12	4.47									
	T8																
	T8				0.79	0.12	2.62	2.19									
	T8				2.25	0.39	4.77	2.96									
	T8																
	T8																
	T8	0.05	2.06	33.09	2.11	0.32	3.53	1.89									
	T8																
	T8	0.06	1.26	14.15	1.76	0.19	2.79	2.83	37.24	5079.65	2346.45	1985.71	60.03	84.67	2.65	357.28	2.43
	T8	0.03	0.76	22.01					72.35	1101.38	1342.92	1395.74	26.05	25.05	1.50	200.62	0.61
	T8	0.04	1.83	33.73													
	T8																
	T8								57.60	2418.04	1407.46	1358.11	7.49	5.02	0.41	12.20	0.14
	T8	0.06	1.88	29.28	0.94	0.23	1.65	1.11	63.19	3802.53	1351.18	2004.15	23.37	27.30	1.15	136.49	0.73
	T8	0.08	1.65	21.88													

* SAM, S-adenosyl-L-methionine; GSH, glutathione (reduced form); GSSG, glutathione disulfide (oxidized form); Put, putrescine; Cad, cadaverine; Spd, spermidine; Spm, spermine; IAA, indole-3-acetic acid; ABA, abscisic acid; SA, salicylic acid; JA, jasmonic acid; IPA, isopentenyl adenine; tZ, *trans*-zeatin; cZ; *cis*-zeatin; tZR, *trans*-zeatin riboside; cZR, *cis*-zeatin riboside; IPA, isopentenyl adenine; C1, C5, C8, lines of regenerants obtained on a control medium; T1, T5, T8, lines of regenerants obtained on a tested media T1 and T5 with Cu^{2+} ions, T8 with Zn^{2+} ions.

Table S4. Descriptive statistics for metAFLP (SV, DMV, DNMV, D_MET and TCIV in CHH, CHG and CG sequence contexts) and biochemical characteristics.

Classification	Variables	Trials	C1	T8	C5	T5	C8	T8
		No. of observations	2	5	21	39	22	48
metAFLP quantitative characteristics (%)	SV	Min*	9.33	6.35	6.10	6.80	7.98	2.09
		Max	10.66	8.73	14.50	12.84	10.05	9.18
		Mean	9.99	7.76	10.46	10.09	8.75	5.43
		Std	0.67	0.78	2.67	1.44	0.45	2.36
	DMV	Min	13.34	12.43	0.88	0.94	1.41	1.06
		Max	13.51	13.21	5.06	2.96	2.93	2.71
		Mean	13.42	12.79	3.36	2.01	2.46	1.88
		Std	0.09	0.27	1.27	0.39	0.37	0.39
	DNMV	Min	6.90	5.03	1.75	1.41	1.79	0.73
		Max	9.42	7.03	7.19	10.86	4.19	4.64
		Mean	8.16	5.99	3.98	4.48	2.74	2.11
		Std	1.26	0.72	1.20	2.07	0.66	0.95
	TCIV	Min	29.74	23.81	9.84	11.70	12.07	4.18
		Max	33.42	28.04	23.55	24.05	16.13	15.73
		Mean	31.58	26.54	17.79	16.59	13.95	9.42
		Std	1.84	1.57	4.46	2.71	0.95	3.22
	D_MET	Min	-6.61	-7.41	-1.89	-0.71	-0.70	-0.90
		Max	-3.92	-5.64	3.41	8.28	2.13	2.85
		Mean	-5.26	-6.79	0.62	2.47	0.28	0.23
		Std	1.35	0.64	1.45	2.03	0.67	0.87
	CHH_SV	Min	2.59	1.66	1.49	1.22	3.30	0.78
		Max	3.12	2.60	4.75	4.36	4.00	3.63
		Mean	2.85	1.98	2.88	2.96	3.63	2.13
		Std	0.27	0.35	0.91	0.76	0.18	0.92
	CHH_DMV	Min	3.98	3.45	0.27	0.09	0.57	0.23
		Max	4.21	4.27	2.03	1.17	1.50	1.20
		Mean	4.09	3.93	1.15	0.61	1.05	0.71
		Std	0.11	0.30	0.49	0.25	0.21	0.21
	CHH_DNMV	Min	1.95	1.06	0.46	0.27	0.30	0.31
		Max	3.47	1.69	2.97	3.78	2.68	2.38
		Mean	2.71	1.30	1.36	1.51	0.81	0.88
		Std	0.76	0.25	0.65	0.96	0.53	0.53
	CHH_TCIV	Min	8.16	6.09	3.20	2.39	4.72	1.86
		Max	10.35	8.20	7.41	8.04	6.82	6.65
		Mean	9.25	6.91	5.25	4.96	5.38	3.66
		Std	1.09	0.75	1.40	1.33	0.47	1.19

	**CHH_dMET**	Min	-2.03	-3.11	-1.18	-0.84	-0.96	-0.69
		Max	-0.73	-2.31	1.75	3.02	1.96	2.04
		Mean	-1.38	-2.62	0.21	0.89	-0.25	0.17
		Std	0.65	0.30	0.80	1.02	0.66	0.59
	**CHG_SV**	Min	4.06	2.38	2.04	1.36	1.94	1.04
		Max	5.13	4.20	4.23	4.29	3.38	3.72
		Mean	4.60	3.15	3.24	3.19	2.64	2.13
		Std	0.53	0.65	0.65	0.79	0.39	0.56
	**CHG_DMV**	Min	5.04	5.00	0.49	0.61	0.35	0.26
		Max	5.49	5.71	2.33	1.60	1.36	1.28
		Mean	5.27	5.40	1.73	1.21	0.83	0.72
		Std	0.22	0.29	0.54	0.24	0.23	0.27
	**CHG_DNMV**	Min	2.35	2.29	0.15	0.48	0.69	0.00
		Max	2.82	3.17	2.44	3.43	1.88	1.40
		Mean	2.58	2.63	1.51	1.50	1.44	0.64
		Std	0.24	0.29	0.62	0.66	0.35	0.30
	**CHG_TCIV**	Min	11.84	10.32	3.20	3.46	3.41	1.31
		Max	13.00	11.91	8.71	8.11	6.67	5.07
		Mean	12.42	11.14	6.44	5.86	4.95	3.51
		Std	0.58	0.58	1.42	0.91	0.82	0.97
	**CHG_dMET**	Min	-3.15	-3.39	-1.47	-0.89	-0.10	-0.51
		Max	-2.23	-1.83	1.22	2.08	0.92	0.65
		Mean	-2.69	-2.78	-0.23	0.29	0.61	-0.08
		Std	0.46	0.54	0.66	0.64	0.27	0.26
	**CG_SV**	Min	2.42	1.93	2.33	3.32	2.14	0.26
		Max	2.68	3.65	5.74	4.85	3.06	3.03
		Mean	2.55	2.63	4.34	3.94	2.48	1.18
		Std	0.13	0.66	1.34	0.42	0.24	1.05
	**CG_DMV**	Min	4.04	2.94	0.00	0.00	0.35	0.11
		Max	4.09	3.84	0.98	0.53	0.85	0.75
		Mean	4.07	3.46	0.47	0.19	0.58	0.45
		Std	0.03	0.32	0.33	0.17	0.14	0.15
	**CG_DNMV**	Min	2.61	1.59	0.26	0.26	0.32	0.00
		Max	3.13	2.52	2.34	3.65	1.53	1.58
		Mean	2.87	2.07	1.11	1.48	0.49	0.59
		Std	0.26	0.35	0.58	0.84	0.27	0.40
	**CG_TCIV**	Min	9.74	7.41	2.93	4.26	3.19	0.78
		Max	10.08	10.21	8.49	8.65	4.84	4.80
		Mean	9.91	8.49	6.11	5.77	3.63	2.25
		Std	0.17	0.96	1.95	1.04	0.36	1.37
	**CG_dMET**	Min	-1.43	-1.71	-0.33	0.00	-0.34	-0.56
		Max	-0.96	-1.14	1.82	3.63	0.68	1.32

			Mean	-1.19	-1.40	0.64	1.28	-0.09	0.14
			Std	0.24	0.21	0.60	0.85	0.26	0.47
Growth and Stress-Related Metabolites	Sulfur-containing metabolites (µmol g^{-1})		No. of observations	2	4	17	29	17	28
		**S-adenosyl-L-methionine (SAM)**	Min	0.037	0.015	0.013	0.010	0.016	0.003
			Max	0.063	0.100	0.082	0.045	0.095	0.076
			Mean	0.050	0.047	0.033	0.026	0.050	0.041
			Std	0.013	0.033	0.018	0.010	0.020	0.018
		**Glutathione (reduced form) (GSH)**	Min	1.890	0.585	0.728	0.631	0.679	0.546
			Max	5.830	2.735	7.908	2.441	2.969	2.336
			Mean	3.860	1.615	2.435	1.466	1.508	1.392
			Std	1.970	0.772	1.719	0.556	0.612	0.474
		**Glutathione Disulfide (oxidized form) (GSSG)**	Min	10.999	5.659	11.371	5.054	10.239	4.966
			Max	29.026	41.129	91.795	45.766	50.579	45.552
			Mean	20.012	25.156	31.365	18.422	24.641	22.907
			Std	9.014	13.012	18.550	9.233	10.196	9.567
	Polyamines (µg g^{-1})		No. of observations	1	3	18	33	20	36
		**Putrescine (Put)**	Min	4.09	1.02	0.41	0.17	0.71	0.79
			Max	4.09	3.63	14.98	34.33	14.96	3.75
			Mean	4.09	2.23	2.56	2.72	4.58	2.06
			Std	0.00	1.08	3.17	5.81	4.93	0.75
		**Cadaverine (Cad)**	Min	0.19	0.25	0.03	0.04	0.09	0.04
			Max	0.19	1.03	0.26	0.44	0.46	0.39
			Mean	0.19	0.52	0.15	0.16	0.19	0.16
			Std	0.00	0.36	0.06	0.10	0.09	0.08
		**Spermidine (Spd)**	Min	7.37	1.76	1.01	0.41	0.82	1.51
			Max	7.37	4.02	37.36	6.24	45.06	5.04
			Mean	7.37	2.82	4.46	2.40	12.29	3.26
			Std	0.00	0.93	8.09	1.60	15.01	0.87
		**Spermine (Spm)**	Min	12.85	0.94	3.08	1.11	1.78	1.11
			Max	12.85	2.81	41.28	13.30	41.69	7.59
			Mean	12.85	1.98	7.91	6.01	11.75	3.08
			Std	0.00	0.78	8.26	2.72	13.92	1.34
	**Ph yt oh [illegible]**		No. of observations	1	1	8	7	10	9

	**Indole-3-acetic acid (IAA)**	Min	27.93	31.53	18.01	24.68	27.37	37.24
		Max	27.93	31.53	175.03	84.90	176.87	413.27
		Mean	27.93	31.53	67.62	48.45	91.44	109.39
		Std	0.00	0.00	62.70	18.50	49.41	109.42
	**Abscisic acid (ABA)**	Min	1368.52	160.40	32.29	118.58	301.42	313.83
		Max	1368.52	160.40	2481.87	1513.76	3430.25	5079.65
		Mean	1368.52	160.40	982.04	648.20	986.53	1975.28
		Std	0.00	0.00	812.28	441.97	867.48	1584.22
	**Salicylic Acid (SA)**	Min	1051.14	1636.59	284.07	1071.79	657.30	1342.92
		Max	1051.14	1636.59	2403.13	3527.77	2491.62	2413.18
		Mean	1051.14	1636.59	1317.80	1853.34	1482.30	1732.32
		Std	0.00	0.00	754.15	801.94	576.48	419.55
	**Jasmonic Acid (JA)**	Min	1240.42	566.45	216.15	362.52	695.05	471.48
		Max	1240.42	566.45	1538.67	968.56	1692.27	2996.88
		Mean	1240.42	566.45	780.84	734.31	1251.81	1411.75
		Std	0.00	0.00	490.82	180.07	259.78	763.91
	***trans*-zeatin (tZ)**	Min	10.67	8.60	5.99	7.79	6.24	2.80
		Max	10.67	8.60	34.53	44.72	38.23	60.15
		Mean	10.67	8.60	14.94	23.08	21.17	34.41
		Std	0.00	0.00	8.45	12.15	10.63	20.26
	***cis*-zeatin (cZ)**	Min	27.24	44.12	3.54	5.56	4.05	2.52
		Max	27.24	44.12	32.63	69.13	41.81	84.67
		Mean	27.24	44.12	12.80	22.97	18.89	28.18
		Std	0.00	0.00	8.21	20.80	9.77	26.74
	***trans*-zeatin riboside (tZR)**	Min	1.02	0.36	0.34	0.21	0.26	0.41
		Max	1.02	0.36	1.19	2.82	13.61	8.07
		Mean	1.02	0.36	0.71	0.82	2.47	2.90
		Std	0.00	0.00	0.33	0.85	3.81	2.34
	***cis*-zeatin riboside (cZR)**	Min	9.32	23.18	5.15	7.46	23.18	5.09
		Max	290.10	23.18	357.28	348.75	23.18	168.03
		Mean	88.14	23.18	119.48	66.67	23.18	42.63
		Std	99.98	0.00	111.76	108.30	0.00	45.26
	**Isopentenyl adenine (IPA)**	Min	0.63	0.78	0.11	0.26	0.14	0.14
		Max	0.63	0.78	1.02	2.94	8.62	2.43
		Mean	0.63	0.78	0.50	0.76	1.20	0.61

	Std	0.00	0.00	0.26	0.90	2.48	0.67

* Min – minimum; Max –maximum; Std –standard deviation; C1, C5, C8, lines of regenerants obtained on a control medium; T1, T5, T8, lines of regenerants obtained on a tested media T1 and T5 with Cu^{2+} ions, T8 with Zn^{2+} ions.
